# Predicting candidate miRNAs for targeting begomovirus to induce sequence-specific gene silencing in chilli plants

**DOI:** 10.3389/fpls.2024.1460540

**Published:** 2024-09-23

**Authors:** Vineeta Pandey, Aarshi Srivastava, Akhtar Ali, Ramwant Gupta, Muhammad Shafiq Shahid, Rajarshi Kumar Gaur

**Affiliations:** ^1^ Department of Biotechnology, Deen Dayal Upadhyaya Gorakhpur University, Gorakhpur, Uttar Pradesh, India; ^2^ Department of Biological Science, The University of Tulsa, Tulsa, OK, United States; ^3^ Department of Botany, Deen Dayal Upadhyaya Gorakhpur University, Gorakhpur, Uttar Pradesh, India; ^4^ Department of Plant Sciences, College of Agricultural and Marine Sciences, Sultan Qaboos University, Al-khoud, Oman

**Keywords:** begomovirus, chilli leaf curl disease, chilli, miRNA, miRNA-mRNA interaction, target prediction algorithms

## Abstract

The begomoviruses are the most economically damaging pathogens that pose a serious risk to India’s chilli crop and have been associated with the chilli leaf curl disease (ChiLCD). Chilli cultivars infected with begomovirus have suffered significant decreases in biomass output, negatively impacting their economic characteristics. We used the C-mii tool to predict twenty plant miRNA families from SRA chilli transcriptome data (retrieved from the NCBI and GenBank databases). Five target prediction algorithms, i.e., C-mii, miRanda, psRNATarget, RNAhybrid, and RNA22, were applied to identify and evaluate chilli miRNAs (microRNAs) as potential therapeutic targets against ten begomoviruses that cause ChiLCD. In this study, the top five chilli miRNAs which were identified by all five algorithms were thoroughly examined. Moreover, we also noted strong complementarities between these miRNAs and the AC1 (REP), AC2 (TrAP) and betaC1 genes. Three computational approaches (miRanda, RNA22, and psRNATarget) identified the consensus hybridization site for CA-miR838 at locus 2052. The top predicted targets within ORFs were indicated by CA-miR2673 (a and b). Through Circos algorithm, we identified novel targets and create the miRNA-mRNA interaction network using the R program. Furthermore, free energy calculation of the miRNA-target duplex revealed that thermodynamic stability was optimal for miR838 and miR2673 (a and b). To the best of our knowledge, this was the first instance of miRNA being predicted from chilli transcriptome information that had not been reported in miRbase previously. Consequently, the anticipated biological results substantially assist in developing chilli plants resistant to ChiLCD.

## Introduction

The begomovirus genus in the family *Geminiviridae* is the most well-known and biggest plant virus genus that comprises ~445 species and causes severe losses to economically important crops (Fiallo-Olivé et al., 2021). Begomoviruses consist of circular ssDNA of either monopartite, which is about 2.7 kb with DNA-A alone, or bipartite genomes comprised of both DNA-A and DNA-B of 2.5–2.6 kb, encapsulated in twinned particles and are predominantly vectored by whiteflies (*Bemisia tabaci*) persistently (Gnanasekaran et al., 2019; Zerbini et al., 2017). Many pathogens, especially viruses, have a considerable impact on chilli farming (Pandey et al., 2021; Mishra et al., 2020), which is primarily restricted to tropical and temperate countries mainly grown for spices, fresh vegetables, etc. According to the FAOSTAT statistical report for the year 2021, chilli is grown on 0.683 million hectares annually and yields 48.39 lakh tonnes. India is the largest producer of chilli with 1.98 million tonnes and contributes 43% of the world’s chilli production. As per the literature, >166 viruses are known to infect chilli (Ali and Gaur, 2024) including begomovirus. The chilli plant infected with begomovirus showed various symptoms, including mottling, mosaic, vein yellowing of leaves, stunting, curling, distortion, flower abortion, and too small unusable fruits (Rojas et al., 2018). Chilli leaf curl virus (ChiLCV) causes Chilli leaf curl disease (ChiLCD) and has been reported as one of the potentially harmful and destructive begomoviruses (Senanayake et al., 2012). Globally, one of the biggest challenges to chilli production has been the worldwide spread of plant begomovirus disease (Varma and Malathi, 2003).

These begomoviral diseases are becoming a threat to global food security (Kumar, 2019), thus demanding the creation of intervention strategies for the successful management of the virus. To date, a number of miRNAs have been discovered in plants and other organisms that regulate disease resistance signalling pathways (Zhang et al., 2023). MiRNAs are tiny molecules of non-coding RNA that play an important part in regulating how genes are expressed. RNA polymerases II and III transcribe miRNA. The resulting precursors are subjected to a set of cleavages to yield mature miRNA. Two nuclear and cytoplasmic cleavage events make up the traditional biogenesis route. More biogenesis routes, however, differ in terms of the quantity and relevance of cleavage events. It is unclear how miRNA precursors are assigned to the various pathways, although the miRNA’s origin, sequence, and thermodynamic stability are significant considerations. They are effective regulators of several biological processes, such as cell division, growth, and apoptosis. The genetic resources found in these miRNAs could be utilised in molecular breeding and to increase disease resistance in agriculture crops (Zhang et al., 2023). Plants resistant to several virus species have been created as a result of the use of RNAi-mediated gene silencing through artificial miRNA (amiRNA) including cucumber green mottle mosaic virus (Miao et al., 2021; Liang et al., 2019), rice stripe virus (Zhou et al., 2022), turnip mosaic virus (Lafforgue et al., 2013; Niu et al., 2006), plum pox virus (Simón-Mateo and García, 2006), cucumber mosaic virus (Duan et al., 2008), potato virus Y (PVY) (Jiang et al., 2011), cotton leaf curl Kokhran virus-Burewala (Ali et al., 2013), cymbidium mosaic virus, and odontoglossum ringspot virus (Petchthai et al., 2018). Transgenic plants with amiRNA-based resistance to *Cucumber Mosaic Virus* (CMV) infection excelled in those strategies with short hairpin RNA-based silence (Duan et al., 2008).

The current investigation’s computational strategy aimed to predict the most efficient miRNAs for begomovirus control. We used computational techniques to anticipate chilli-derived miRNA targets in the begomovirus genome. This study updates the miRNA synthesis in the chilli host using the C-mii tool. Plant miRNAs provided by the host can suppress gene expression levels by cleaving their mRNA targets (Ali et al., 2013). Chilli miRNAs were analysed to better understand host-virus interactions. We used five miRNA target prediction algorithms i.e., C-mii, miRanda, psRNATarget, RNAhybrid, and RNA22 to validate the interaction between miRNA and begomovirus causes ChiLCD. We also ensured the thermodynamic stability of the miRNA-miRNA duplex. This study revealed siRISC-sensitive cleavage sites in the begomovirus genome to create viable amiRNAs that will be further used to silence a specific viral sequence. amiRNA constructions are highly selective in silencing the target gene, resulting in minimal off-target consequences. Using this technology, begomovirus resistance can be developed in chilli cultivars, which will offers stability, environmental safety, and excellent specificity, making it an effective method. The silenced expression was stably communicated to future generations (Niu et al., 2006). The predicted miRNA may transform chillies to produce begomovirus-resistant plants.

## Materials and methods

### Sequence retrieval, assembly and quality check of chilli biological data

The transcriptome data of chilli (SRR595054) for leaf tissue samples was filtered and retrieved from a public database (https://www.ncbi.nlm.nih.gov/sra). According to the NCBI database, the experimental condition followed the procedures listed below: RNA-seq of chilli from entire leaf tissue was performed using the Illumina HiSeq-3000 for mRNA sequencing. The downloaded data were processed, filtered, and assembled using rnaviralSPAdes (*de novo* assembler for transcriptomes, metatranscriptomes, and metaviromes (Bushmanova et al., 2019) using Galaxy Version 3.15.4 (https://usegalaxy.org/), a web server. On the same web server, the QUAST program statistically analysed the processed assembly data (Gurevich et al., 2013).

### miRNA prediction and its secondary structure

C-mii v1.11 was utilised to detect miRNAs, their secondary structure, and targets in chilli (Numnark et al., 2012). The putative miRNA candidates were compared to the published miRNAs of all plants in miRBase using BLASTN (e-value: 1e-8). To remove protein-coding sequences, we used BLASTX (e-value < 1e−20) against the protein databases UniProtKB/Swiss-Prot (plant only) (version 2010_12) and UniProtKB/TrEMBL (release 2011_01). UNAFold was used to fold both the primary and precursor miRNAs. The UNAFold parameters used were a maximum base pair distance of 3000, a maximum bulge/interior loop size of 30, and a single thread run at 37°C. The C-mii identified miRNAs using the criterion that predicted miRNAs should be between 19 and 25 nucleotides in length; only four substitutions were permitted for predicted mature miRNAs for a known miRNA; mature miRNA localization within the stem-loop structure with one arm; and the secondary structure has a negative minimal folding free energy (MFE) and a high MFE index (MFEI). The MFEI of the pre-miRNAs individually were assessed by the RNAfold web server (http://rna.tbi.univie.ac.at/cgi-bin/RNAWebSuite/RNAfold.cgi) and C-mii tool.

### Retrieval of target genome sequence (begomovirus)

The targets for predicted miRNA used in this study were whole genome sequences of begomovirus including ten DNA-A, associated ten betasatellites and one alphasatellite that we identified during the study of Chilli leaf curl disease (ChiLCD) across different regions of India (**Supplementary Table 1**). These sequences were retrieved from the NCBI GenBank database.

### Target prediction in begomovirus genome

One important component in determining reliable miRNA-mRNA interaction hybridization is target prediction. The optimal miRNA target candidate is currently predicted and identified using a variety of target prediction techniques. Every tool for miRNA-predicting targets employs distinct standards and techniques. To identify the most pertinent chilli miRNAs for begomovirus genome silencing, we evaluated five target prediction methods that have been reported in the literature: cmii, miRanda, RNA22, RNAhybrid, and psRNATarget (**Table 1**). Complementarity-based miRNA-mRNA binding is computed by these computational techniques. There are two regions in this binding: seed and mid. A mismatch in the intermediate region of the miRNA-mRNA association causes less harm than a mismatch in the seed region. This gives rise to the computation’s oversensitivity. To increase the sensitivity of the prediction, we might specify a greater penalty for a mismatch in the seed region. Three distinct prediction levels—individual, union, and intersection—were examined using an efficient computational method to identify miRNA targets. The workflow process in detail is shown in **Supplementary Figure 1**.

**Table 1:** Analysing the unique characteristics of the five target prediction tools.

Tools	Algorithms	Organisms	Seed pairing	Target site accessibility	Multiple sites	Translation Inhibition	Source	Parameter Used
**C-mii**	FASTA file	Plant	Yes	Yes	Yes	Yes	Software (GUI)	BLAST+SCAN e-value =20 Binding Score <= 4
**miRanda**	Local alignment	Human, rat, fly, and worm	Yes	Yes	Yes	Yes	http://www.microrna.org/ (accessed on 20 July 2023)	Free energy = −15 Score threshold = 140, Kcal/mol, Gap Extend penalty = −4.00 Gap Open penalty = −9.00,
**RNAhybrid**	Interamolecular hybridization	Any	Yes	Yes	Yes	Yes	http://bibiserv.techfak.unibielefeld.de/rnahybrid (accessed on 30 July 2023)	Hit per target = 1
**psRNATarget**	Smith-Waterman	Plant	–	Yes	Yes	Yes	https://www.zhaolab.org/psRNATarget/ analysis?function=2 (accessed on 1 August 2023)	Expectation score = 7, HSP size = 19 Penalty for G:U pair = 0.5 Penalty for opening gap = 2
**RNA22**	FASTA	Human, mouse, fly, and worm	–	Yes	Yes	–	https://cm.jefferson.edu/rna22/Interactive/ (accessed on 20 August 2023)	Sensitivity= 63%, Specificity = 61% GU region allowed in seed region= no limit MFE for heterduplex= −12

### C-mii

According to previous research, the majority of plant miRNAs have perfect or near-perfect sequence complementarity when they bind to their targets (Llave et al., 2002; Reinhart et al., 2002). It assists in the control of post-transcriptional expression of genes by translation inhibition and cleavage (Yu et al., 2017). The selection of miRNA targets in plants using homologous miRNA search becomes feasible by this event, which offers an effective strategy. C-mii developed the five criteria for predicting miRNA and target genes i.e., there should be no more than six mismatches among predicted mRNAs and target genes; target sequences with only one mismatch at each of places 1–9 in the miRNA binding site; two consecutive mismatches not more than two and none at positions 10 or 11; the number of G: U pairs between miRNA and its potential target should not more than 5 and; MFE of the target duplex and miRNA must be negative (**Table 1**).

### miRanda

The most widely used standard computational algorithm for miRNA-target prediction is miRanda. Predicting host-virus interactions involves several computational aspects. According to John et al. (2004), some characteristics followed by this versatile algorithm include minimal free energy (MFE), cross-species target conservation, RNA-RNA duplex dimerization, miRNA target duplexes, seed-based interactions, and sequence compatibility. The source website provided a C programming language version of the miRanda algorithm. The default parameters were used to run the miRanda algorithm (**Table 1**).

### psRNATarget

Using a web server, users can access the highly sensitive miRNA prediction tool in plants known as the psRNATarget algorithm. The target viral mRNA region and host miRNAs are reversely complementary in the psRNATarget algorithm, which can be accessed at http://plantgrn.noble.org/psRNATarget/ (Dai and Zhao, 2011). Using the psRNATarget methodology, target-site accessibility is assessed by computing the unpaired energy (UPE). Using user-specified parameters and an expected cut-off value of 7, the interaction of miRNA and mRNA was calculated (**Table 1**).

### RNA22

Target sites with adequate hetero-duplexes may be predicted using RNA22, a novel pattern-recognition algorithm that is simple to use and available online (http://cm.jefferson.edu/rna22v1.0/). Pattern recognition, MFE, non-seed-based interactions, and site complementarity are some of the most sensitive algorithmic elements (Miranda et al., 2006). This method (Loher and Rigoutsos, 2012) does not consider cross-species conservation filters. The study was carried out using default settings (**Table 1**).

### RNAhybrid

A new, versatile online tool called RNAhybrid makes it simple and quick to determine miRNA targets. A key aspect is the hybridization of mRNA and miRNA based on MFE. According to Krüger and Rehmsmeier (2006), additional features include seed match, helix limitations, free energy, target-site abundance, and site complementarity. It’s an online tool for quick miRNA target prediction by mRNA and miRNA MFE hybridization. The parameters that were set as default were selected (**Table 1**).

### Target annotation of predicted CA-miRNA

In C-mii, the function and gene ontology (GO) are provided for potential targets via the target annotation module. This allows us to adjust the E-value and BLASTX hit count while selecting a protein database. For annotation, we have selected only those predicted miRNAs that showed maximum targets against the ten begomoviruses through at least four algorithms. This has helped to make the data more understandable and presentable for annotation. Predicted miRNAs were subjected to target search against selected family contig sequences of all plant-source mature miRNA from miRbase under the default parameter. All of the chosen contig sequences were searched for in the complementary position of the anticipated miRNAs using target scanning. C-mii performed functional annotation of putative target transcripts using the UniProt/Swiss-Prot (plants only) databases. C-mii additionally assessed the regulation of identified miRNAs in secondary metabolic, biological, molecular, and cellular process pathways. Cytoscape version 3.1 was used to visualise the biological network of the top five predicted miRNAs and their targets chilli protein along with the MFE value for each interaction (Su et al., 2014).

### CA-miRNA–target interaction mapping

Using the R program, an interaction map between begomovirus ORFs and chilli miRNAs was generated by applying the Circos algorithm (Krzywinski et al., 2009).

### Thermodynamic stability: free energy evaluation of duplex binding

Sequence alignment is undoubtedly useful in predicting miRNA–mRNA interactions, but the thermodynamic characteristics of the miRNA–mRNA complexes offer crucial indications for assessing hybridization stability (Riolo et al., 2020). The free energy (ΔG) of the anticipated interaction is used in most miRNA target prediction systems to evaluate the thermodynamic parameters of the miRNA-mRNA complex. RNAcofold, a new web-based server (http://rna.tbi.univie.ac.at/cgi-bin/RNAWebSuite/RNAcofold.cgi), is used to estimate the heterodimer free energy (ΔG) related to interactions among miRNA and mRNA (Bernhart et al., 2006). Using the miRNA-target duplex from psRNATarget, the appropriate begomovirus target genomic sequences and chilli miRNAs were analysed using the default settings of the RNAcofold web server.

## Result

### miRNA prediction

**Figure 1:**
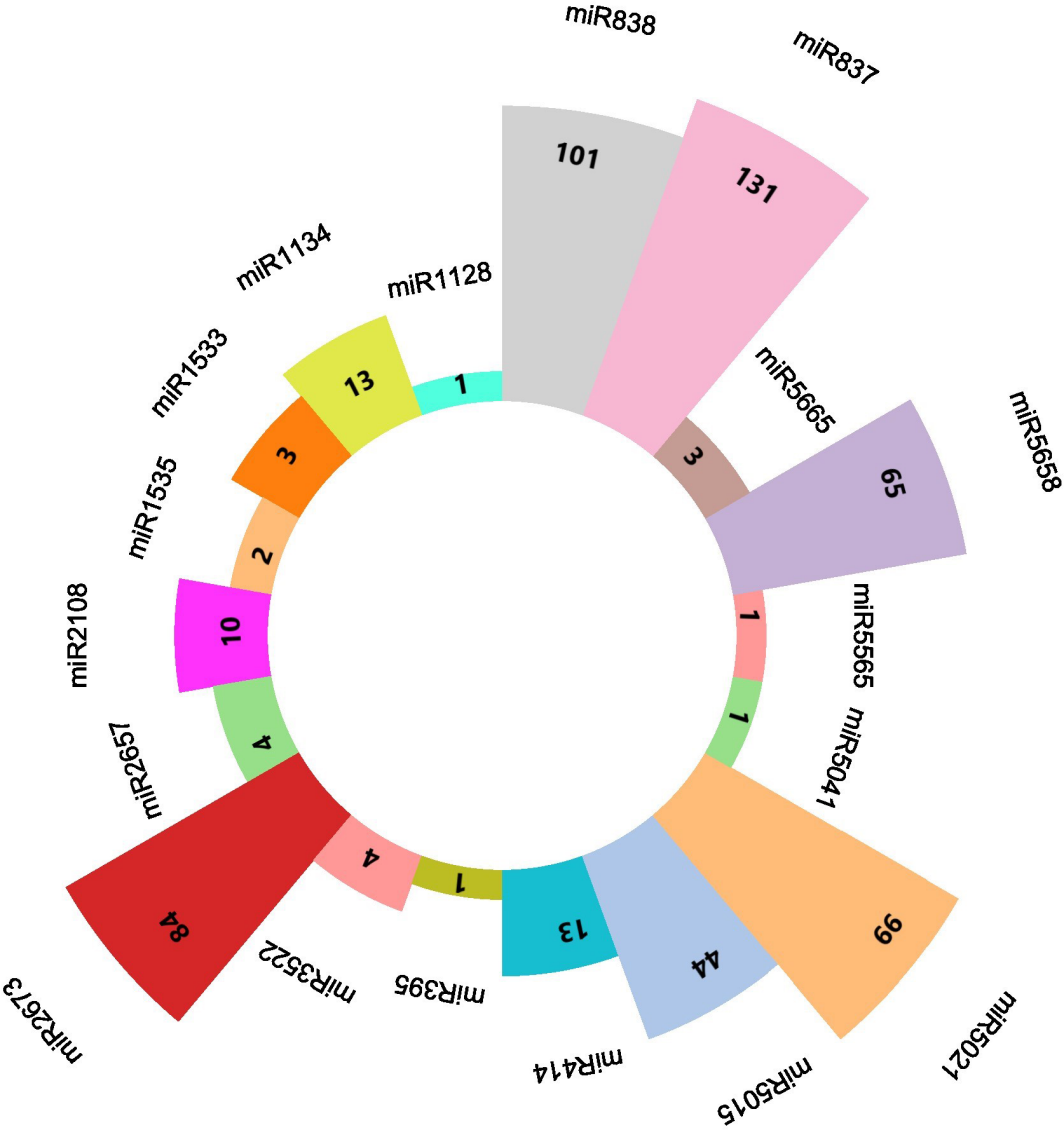
A diagrammatic representation of every predicted miRNA from the C-mii tool shows how many transcripts there were all together for each miRNA.

**Figure 2:**
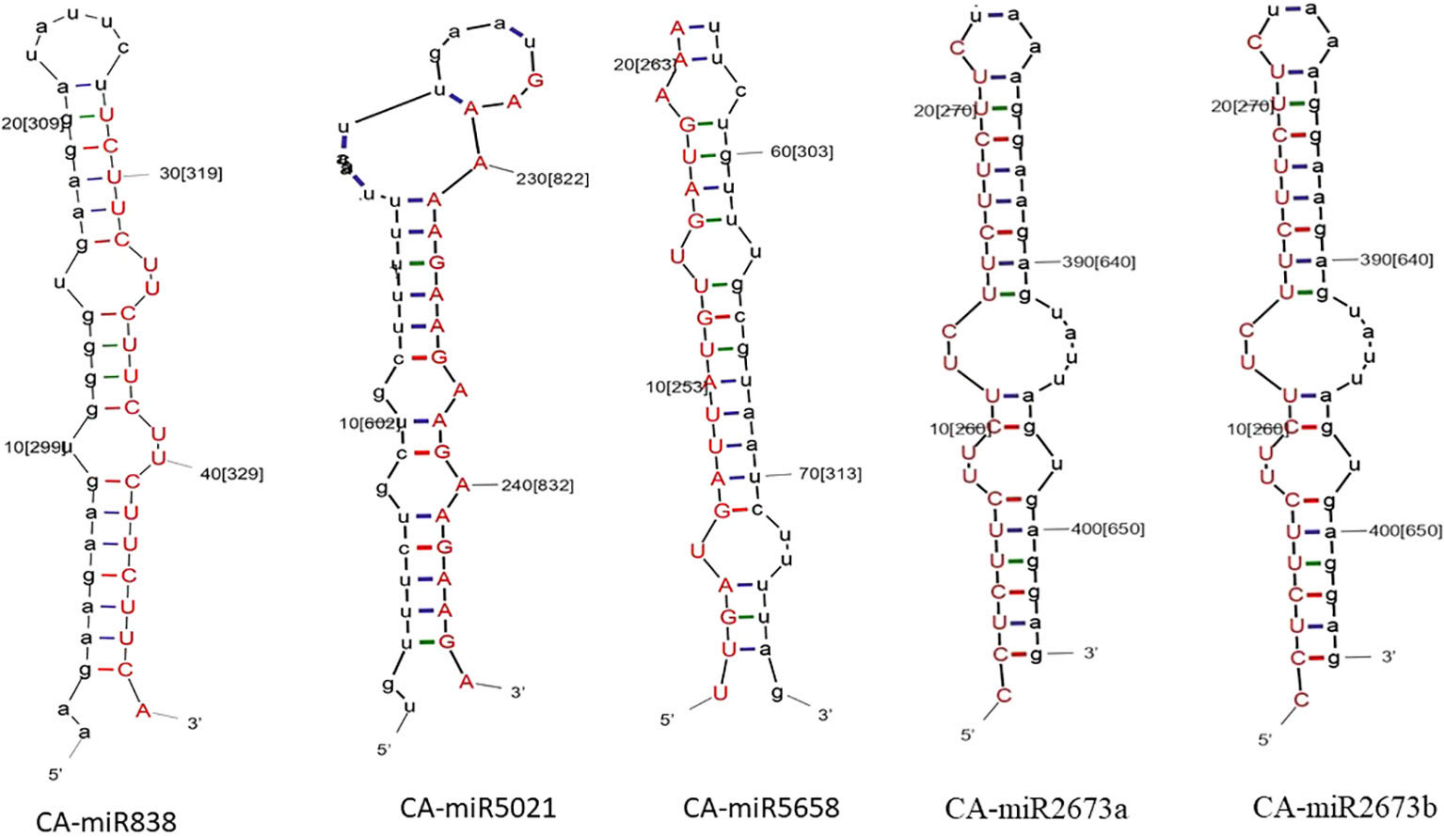
The secondary structure of the top five predicted miRNA members is established by all five algorithms: the red strand indicates the predicted miRNA strand, while the black strand indicates the target in the chilli genome.

The quality check of large chilli transcriptomic data is listed in **Supplementary Table 2** with a shorter contig length and 41.40% GC content, which added consistency to the data with no N’s per 100 kbp from the SRA data through the QUAST tool (Reinhart et al., 2002). A total of 580 miRNAs were predicted in the C-mii tool (**Figure 1**). Subsequently, 36 sequences were chosen as miRNA candidates after careful consideration of the homology and secondary structure prediction results. Besides MFEIs (<-0.5), we filtered the mature miRNA findings by restricting the number of two-nucleotide 3’ overhangs up to <=1, the number of mismatches, the number of bulges (<=0), and bulge sizes (<=0). This predicted sequence belongs to 20 distinct families, which have 22 members (**Table 2**). The majority of the miRNA families identified comprised only one member, except miR2657 and miR2673 which have two members. In the instance of a few members i.e., CA-miR5021 and CA-miR5658 we identified many transcripts and only single miRNA candidates predicted from a single transcript with the highest MFEI were used to eliminate false positive findings and increase accuracy. The secondary structure of predicted miRNA was also indicated in **Figure 2**, which shows miRNA targeting the chilli genome.

**Table 2:** List of recently estimated homologs of chilli miRNAs, mature miRNA sequences, and nucleotide-based characteristics of the precursor miRNA sequences derived from SRA data using the C-mii program.

Predicted miRNA family	Homolog miRNA	LP	LM	NM	Strand	MFE (kcal/mol)	MFEI (kcal/mol)	GC (%)	AU (%)	NC	Predicted miRNA sequence
CA-miR837-5p	aly-miR837-5p	72	21	4	+	-15.1	-1.078	19.44	80.56	A(23), U(35), G(9), C(5), N(0)	5':UAUUUUUUCUUUUUUUUUUUA:3'
CA-miR838	aly-miR838	48	21	4	+	-18.4	-1.022	37.5	62.5	A(11), U(19), G(10), C(8), N(0)	5':UCUUCUUCUUCUUCUUCUUCA:3'
CA-miR5021	ath-miR5021	59	20	4	+	-29.4	-1.05	47.45	52.55	A(18), U(13), G(14), C(14), N(0)	5':UAAGAAGAGGAAGAUCAAAA:3'
CA-miR5015b	ath-miR5015b	46	21	3	+	-10.3	-0.572	39.13	60.87	A(10), U(18), G(12), C(6), N(0)	5':UUUGUUGUUGUUGGUGUUUUG:3'
CA-miR5658	ath-miR5658	64	21	4	+	-23.7	-0.79	46.87	53.13	A(15), U(19), G(20), C(10), N(0)	5':AUGAUGAUGAUUAUGGUGAUC:3'
CA-miR5665	ath-miR5665	461	21	4	+	-107.9	-0.544	42.95	57.05	A(116), U(147), G(135), C(63), N(0)	5':GUGGUGGACAAGAUGAGGGAA:3'
CA-miR1127	bdi-miR1127	149	21	4	+	-31.5	-0.684	30.87	69.13	A(53), U(50), G(19), C(27), N(0)	5':AAGUACUCCCUCCGUCCUAAG:3'
CA-miR1134	tae-miR1134	219	24	4	–	-47.1	-0.611	35.15	64.85	A(92), U(50), G(48), C(29), N(0)	5':CGAAAAAAACAAGAAGAAGAAGAU:3'
CA-miR1533	gma-miR1533	69	19	4	–	-9.8	-0.89	15.94	84.06	A(22), U(36), G(4), C(7), N(0)	5':UUAGUAAAAAUAAUAUGGA:3'
CA-miR1535	gma-miR1535	222	19	3	+	-44.2	-0.581	34.23	65.77	A(70), U(76), G(47), C(29), N(0)	5':UUUCUUUGCGGUGAUGUCU:3'
CA-miR2108b	gma-miR2108b	202	21	4	+	-44.4	-0.541	40.59	59.41	A(47), U(73), G(39), C(43), N(0)	5':UUUAUGUUUUGUGUUUGUUAU:3'
CA-miR3522	gma-miR3522	79	19	4	+	-24.1	-0.86	35.44	64.56	A(23), U(28), G(16), C(12), N(0)	5':GGACCAAAUGAGCAGGGAA:3'
CA-miR5041	gma-miR5041	97	21	4	+	-25.1	-0.643	40.2	59.8	A(21), U(37), G(21), C(18), N(0)	5':UUUGGUCUUCAUCUUGCUCAC:3'
CA-miR2657a	mtr-miR2657b	94	22	6	+	-16.8	-0.646	27.65	72.35	A(25), U(43), G(19), C(7), N(0)	5':UGUUAUUUCAUCUUGUUUCUUG:3'
CA-miR2657b	mtr-miR2657a	94	22	6	+	-16.8	-0.646	27.65	72.35	A(25), U(43), G(19), C(7), N(0)	5':UGUUAUUUCAUCUUGUUUCUUG:3'
CA-miR2673a	mtr-miR2673a	404	22	5	+	-93.6	-0.55	42.07	57.93	A(130), U(104), G(92), C(78), N(0)	5':CCUCUUCUUCUUCUUCUUCUUC:3'
CA-miR2673b	mtr-miR2673b	404	22	5	+	-93.6	-0.55	42.07	57.93	A(130), U(104), G(92), C(78), N(0)	5':CCUCUUCUUCUUCUUCUUCUUC:3'
CA-miR395x	osa-miR395x	189	21	4	–	-34.2	-0.51	35.44	64.56	A(72), U(50), G(49), C(18), N(0)	5':GUGAAGUGUUUAGAUUUUCUC:3'
CA-miR414	osa-miR414	517	21	4	+	-135.5	-0.648	40.42	59.58	A(127), U(181), G(107), C(102), N(0)	5':UCAUCCACUUCAGCAUCUUCC:3'
CA-miR5565e	sbi-miR5565e	134	19	5	–	-31.5	-0.552	42.53	57.47	A(33), U(44), G(41), C(16), N(0)	5':CUGUGUGGAUGUUGUCGCG:3'
CA-miR1128	ssp-miR1128	149	21	5	+	-31.5	-0.684	30.47	69.53	A(53), U(50), G(19), C(27), N(0)	5':AAGUACUCCCUCCGUCCUAAG:3'
CA-miR2950	vvi-miR2950	109	21	6	–	-22.9	-0.532	39.44	60.56	A(20), U(46), G(24), C(19), N(0)	5':UUCUUUGUCUUGUACACUGGA:3'

LP, length of precursor; LM, Length of mature miRNA; NC, Nucleotide content; NM, number of mismatches, MFE, minimal folding free energy, MFEI, minimal folding free energy index.

### Characterization of predicted pre-miRNAs

#### Length variation

The majority of plus strands were found for predicted miRNAs. The mature miRNA sequences exhibited nucleotide variations up to 19–24, with a few miRNA families displaying lengths of 19 and 24 nt (**Table 2**). The distribution of A, U, G and C content was not uniform. Comparing the predicted mature miRNA length, precursor miRNAs varied greatly. Significant differences in pre-miRNA length were also noted in previous research (Patanun et al., 2013; Barozai et al., 2012; Wang et al., 2012). The mature chilli miRNAs that were found also had their stand characteristics analysed to determine whether the antisense or sense strands were used in their transcription. The plus strand included seventeen of the known miRNAs, while the negative strand contained five (**Table 2**).

#### miRNA GC content

The creation and stabilisation of the secondary structure of stem-loop hairpins is aided by the coupling of three hydrogen bonds between G and C. By this reasoning, a high percentage of GC content in the sequence should be necessary for the long-term stability of the secondary structure of RNA. GC content is crucial for correct processing and plant abundance (Narjala et al., 2020). In this study, the predicted miRNA family CA-miR5021 showed 47.45% GC in their pre-miRNA sequence. The overall range of GC% varied from 19.44 to 47.45% (**Table 2**). AU content was high when analysed alongside GC content, ranging from 42.75 to 80.56 (**Table 2**).

#### MFE and MFEI

An additional criterion, such as MFE, was used for assessing RNA’s or secondary structure’s stability. According to Bonnet et al. (2004), precursor miRNAs have less folding energy than other non-coding RNAs. The MFE of the 22 anticipated pre-miRNA members ranged between -9.8 to -135.5 kcal/mol. miRNA cannot be sufficiently characterised by MFE alone since precursor miRNAs vary in length. The MFEI value of individual miRNAs was calculated through C-mii to check their stability (**Table 2**). Using MFEI, miRNAs can be differentiated from both coding and non-coding RNAs (Zhang et al., 2006a). The MFEI ranged from -0.51 to -1.078 kcal/mol for each predicted pre-miRNA in the current investigation, with an average of approximately −0.67 kcal/mol (**Table 2**). Compared to rRNAs (0.59), tRNAs (0.64), and mRNAs (0.62–0.66), this is noticeably higher (Zhang et al., 2006a; Singh et al., 2016a). Additionally, we used the RNAFold to check the MFEI value computed in cmii and observed a similarity between the two results, suggesting the thermodynamic stability associated with the secondary structure.

#### miRNA-mediated gene regulatory pathways in chilli plant

**Figure 3:**
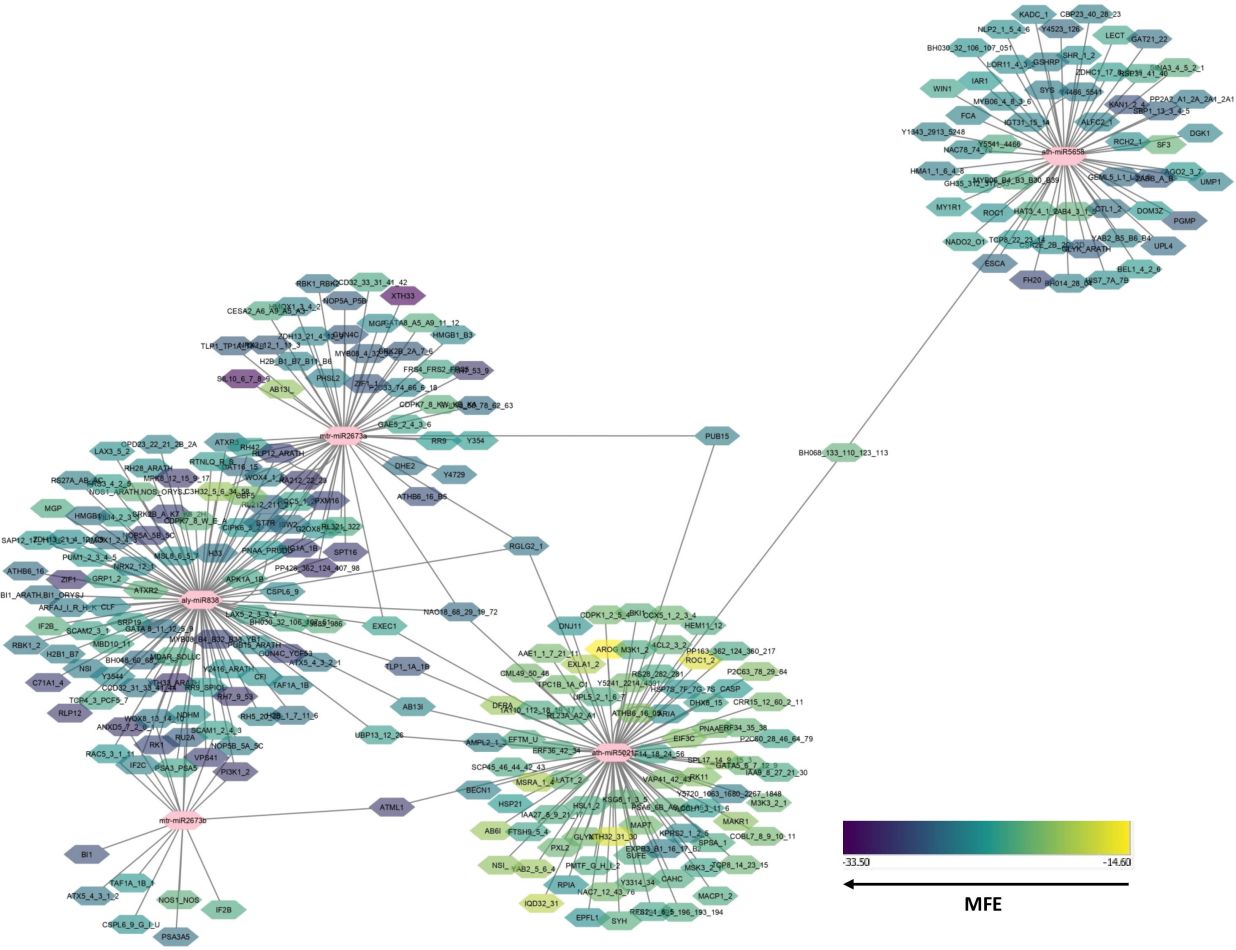
Predicated miRNA families and their related target interactions within chilli were shown in networks by using Cytoscape 3.2 (Shannon et al., 2003). The colour blue indicated all targets, whereas the predicated miRNA families were displayed in ten distinct colours.

Using the application of C-mii, we observed 1258 targets of chilli protein targeted by our 20 predicted miRNA families. Based on the evaluation of the connection distribution network, it was evident that CA-miR5021, CA-miR837-5p, and CA-miR838 have the maximum number of interactions with chilli protein, i.e., 501, 201, and 101 targets, respectively. Out of 1258 targets, seventeen miRNA families—CA-miR5658, CA-1134, CA-miR2108, CA-miR5041, CA-miR3522, CA-miR2657, CA-miR2673, CA-mi3522, CA-mi1533, CA-miR395x, CA-mi1127, CA-2657a, CA-2657b, CA-miR2673a, CA-miR2673b, CA-miR414, CA-miR5565e, CA-miR1128, and CA-miR5041—co-regulated the activity of 445 targets. Considering that all 20 miRNAs that were used to target the chilli protein regulated the process in an extensive amount (Ramesh et al., 2017), we focused on the top CA-miR838, CA-miR5021, CA-miR5658, CA-miR2673a and CA-miR2673b amiRNAs that have the highest number of begomoviral targets that identified at least four target prediction algorithms. This aids in the proper presentation and interpretation of the data. These biological networks of five miRNA and their targets were displayed by using Cytoscape 3.10 (**Figure 3**) (Su et al., 2014; Shannon et al., 2003). The colour intensity in hexagons was used to differentiate the host targets based on the MFE value for each miRNA-host protein target, with higher colour intensity indicating a lower MFE. All of the host proteins showed that these five miRNAs targeted were involved in important biological, metabolic, and cellular processes. During virus infections, these begomoviruses may act as mimics of the miRNA targets and up or down-regulate the function of the proteins that they were targeting in the chilli.

### CA-miRNA target prediction on begomovirus genome and mapping

**Figure 4:**
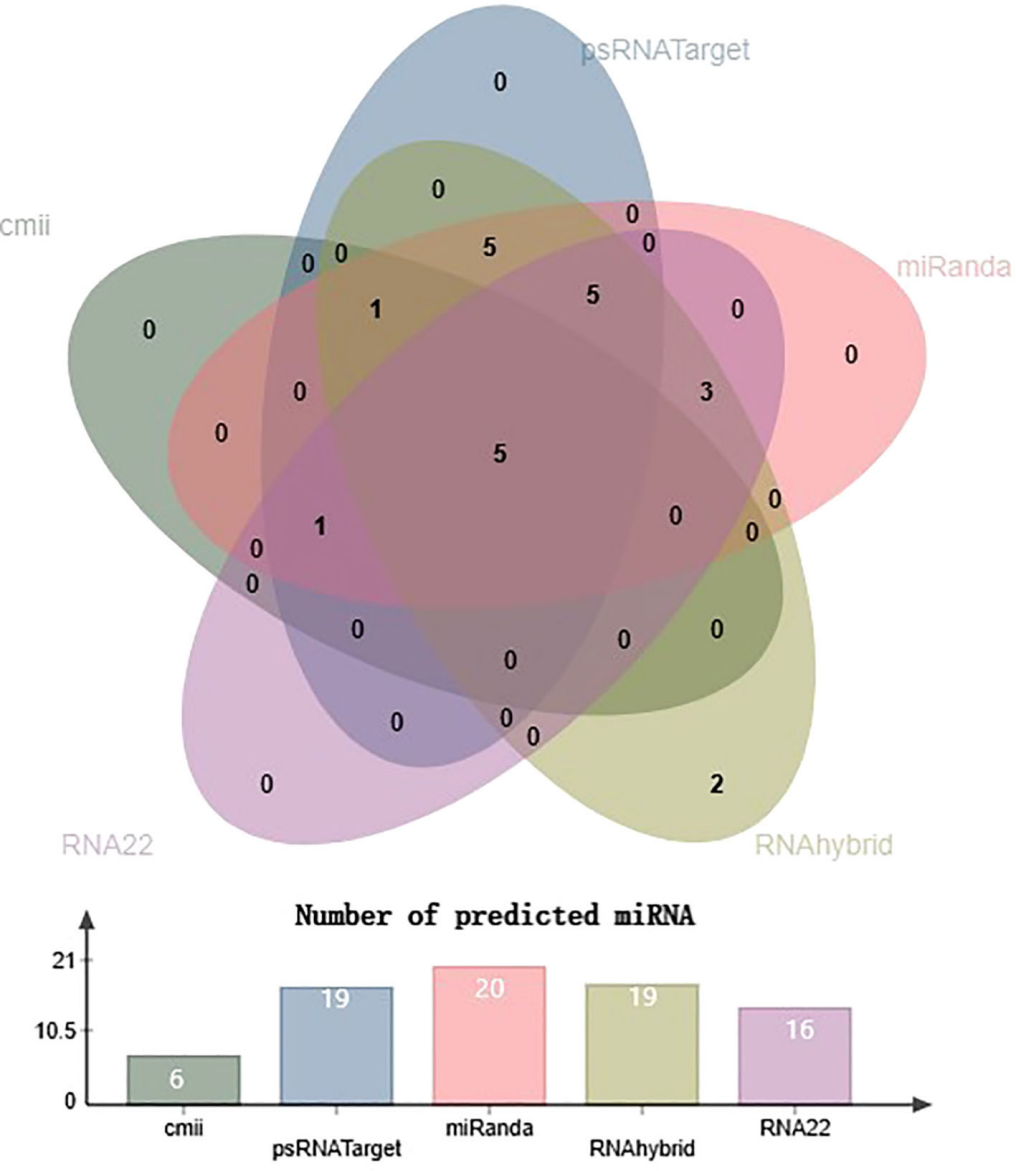
Venn diagram plot of chilli-encoded miRNAs concluded by all five algorithms. Chilli-encoded miRNAs target 148 sites in the genomes of ten distinct isolates of begomovirus. Furthermore, all the computational techniques in this study validate the total number of targeting sites of 22 chilli miRNAs showing interaction with begomovirus. Additionally, all five of the mathematical methods employed in this study predicted the presence of five chilli miRNAs: CA-miR838, CA-miR5658, CA-miR5021, CA-miR2673a, and CA-miR2673b.

**Figure 5:**
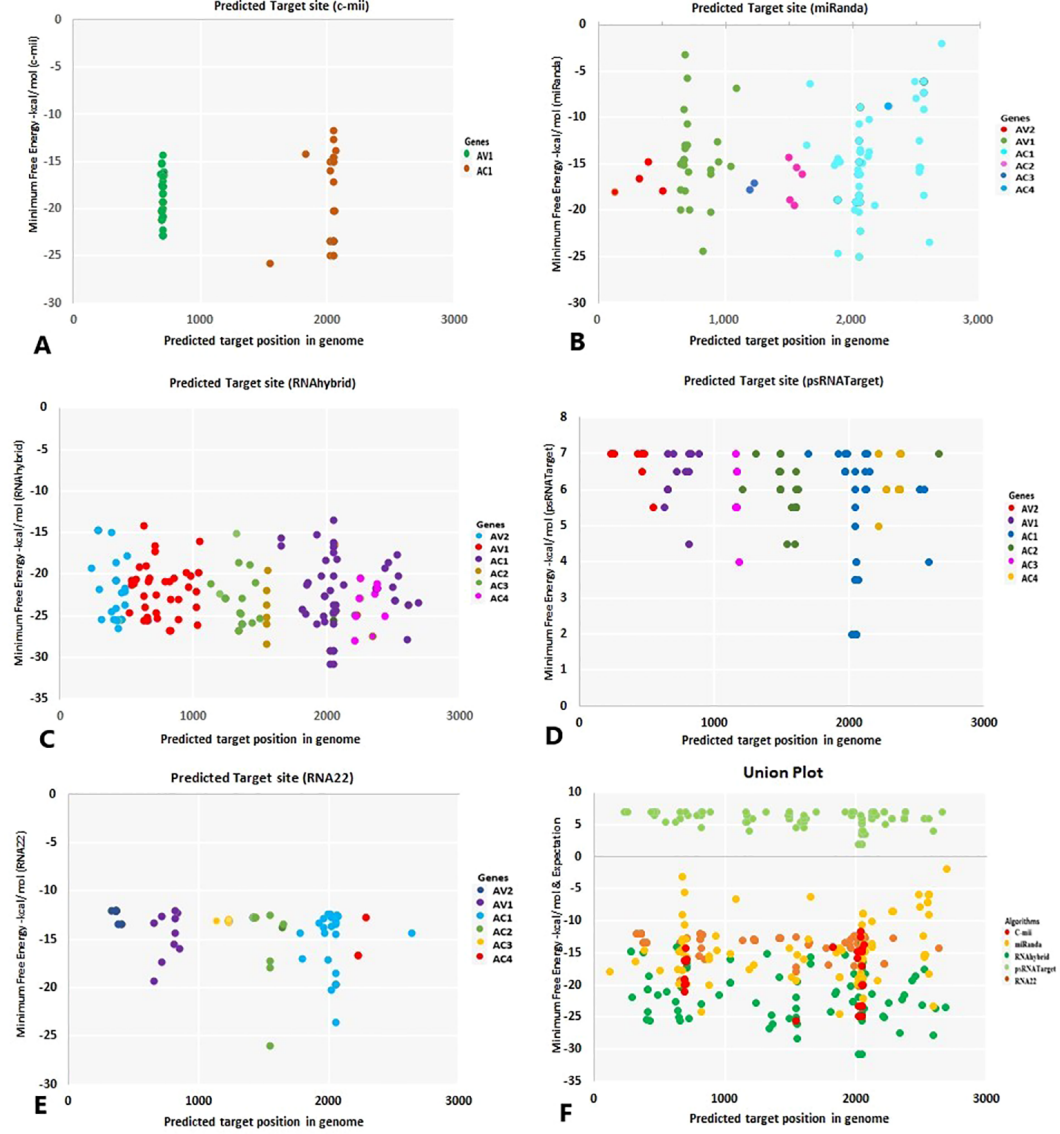
The “five algorithms” method predicted specific chilli CA-miRNAs and their high-confidence binding regions in the begomovirus genome. **(A)** C-mii identified the miRNA regions. **(B)** miRanda reported the miRNA sites of the target. **(C)** Chilli miRNA binding spots were found using RNAhybrid. **(D)** psRNATarget suggests chilli miRNA binding sites. **(E)** RNA22’s prediction of miRNA binding affinity site. **(F)** Union plot illustrating every predicted binding site found by each algorithm that was applied. Several copies of the binding sites for miRNA targets are shown as coloured dots. Different colours denote the begomovirus’s targeted genes.

**Figure 6:**
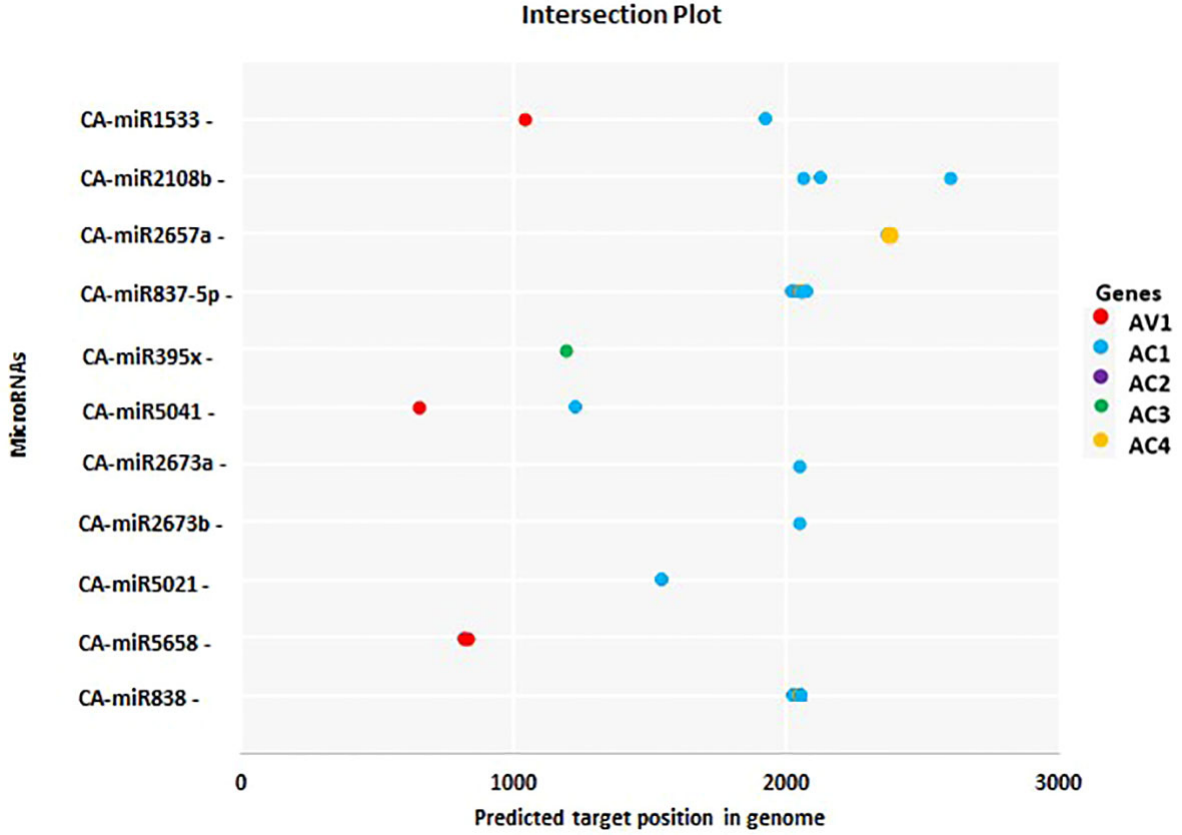
The most common chilli miRNAs determined by at least two different algorithms at homologous loci are represented by an intersection plot. There are colour codes provided in the Figure. Expectation cut-off score (psRNATarget) and minimum free energy (MFE) (cmii, miRanda, RNAhybrid, and RNA22), are provided.

miRNAs that have a perfect or nearly perfect match to their target mRNAs help regulate post-transcriptional gene expression via translation inhibition and cleavage. The targeted mRNA was cleaved when the miRNA and target mRNA sequences were completely complementary. In contrast, partial compatibility often reduces gene expression by inhibiting the translation of the targeted mRNA in the host (Ramesh et al., 2017). This study predicted host miRNAs that can specifically target identified begomovirus isolates in chilli plants, and all 20 of the predicted miRNA families had potential targets. We used the five algorithmic tools—cmii, miRanda, RNA22, psRNATarget, RNA22, and RNAhybrid—to forecast the binding strength as well as the relevance of the 20 chilli potential miRNAs to the begomovirus genome because miRNA binding to target RNA genomes is highly diverse (**Supplementary Figure 3**; **Table 3**). When several *in silico* computational approaches were applied to show target alignment with begomovirus isolates, only the regulation of about 1124 target transcripts was seen for those miRNAs. Five miRNA members (CA-miR838, CA-miR5021, CA-miR5658, CA-miR2673a, and CA-miR2673b) out of the 22 predicted miRNA members were identified by all five algorithms, while seven were supported by four and seven by three algorithms (**Table 3**). Six miRNAs were predicted using cmii, and CA-miR5021 had the highest target value of 24 targets (**Supplementary Tables 3**-**7**). Similarly, 20 miRNAs were reported by MiRanda, while CA-miR2673 (a and b) showed the highest 19 begomovirus targets. Additionally, in RNA22, 16 miRNAs exhibited an affinity for their target, among which miR2673 (a and b) shows the highest affinity for 23 targets within the begomovirus genome. While examining the psRNATarget result, we identified 19 miRNA-targeting virus isolates with 30 targets individually through CA-miR2108b (**Table 3**). In contrast, RNAHybrid shows 17 predicted miRNAs, of which just CA-miR5665 hits 59 loci (**Table 3**). Based on the total number of targeted sites of all predicted CA-miRNA were detected by the union of consensus between the targeting result of multiple algorithms (**Figure 4**) and also predicted the binding sites at particular coding regions of begomovirus (**Figures 5A–F**). Two algorithms verified the consensus hybridization binding regions at the shared locus revealed by twenty-two consensual chilli miRNAs members (**Figure 6**). The consensus hybridization site of CA-miR838 was found at locus 2052 by three computational techniques (miRanda, RNA22, and psRNATarget), additionally, miRanda and cmii predicted a binding site in the same area at locus 2051. Likewise, four algorithms (miRanda, RNA22, psRNATarget, and RNAhybrid) found a locus range from 817-819 for CA-miR5658, while miRanda, RNA22, and C-mii identified the binding affinity of CA-miR5021 at locus 1542 (**Figure 6**; **Supplementary Tables 3**-**7**).

**Table 3:** List of chilli predicted miRNA showing target within begomovirus DNA-A (ORFs), alphasatellite and betasatellite through a different algorithm.

	Algorithms predicted miRNA within DNA-A, Betasatellite and Alphasatellite							
	DNA-A						Betasatellite	Alphasatellite
miRNA	AV2	AV1	AC1	AC2	AC3	AC4	betaC1	Rep
**CA-miR838**	------------	miR, RH	cmii, miR, psRT, RNA22, RH	miR, psRT, RNA22, RH	RH	RNA22, RH	miR, RH	RH
**CA-miR837-5p**	------------	miR	cmii, miR, psRT	psRT	miR	------------	------------	------------
**CA-miR5665**	RH	RNA22, RH	miR, RNA22, RH	RNA22, RH	RNA22, RH	miR, RNA22, RH	miR, RNA22, RH	RH
**CA-miR5015b**	RNA22	miR, psRT	miR, psRT, RNA22, RH	miR, psRT, RH	miR, psRT, RNA22, RH	miR, psRT, RNA22, RH	miR, psRT	miR, psRT
**CA-miR5658**	miR	cmii, miR, psRT, RH	miR, psRT, RNA22, RH	miR, RNA22, RH	miR, psRT, RNA22, RH	psRT, RH	RNA22, RH	RNA22
**CA-miR5565e**	miR, RH	miR, psRT, RNA22, RH	miR, psRT, RNA22, RH	RNA22, RH	miR, RNA22, RH	RNA22, RH	miR, RNA22, RH	RH
**CA-miR5041**	PsRT, RH	miR	miR, psRT, RNA22, RH	RNA22, RH	RH	RNA22, RH	miR, RH	RH
**CA-miR5021**	------------	miR	cmii, miR, psRT, RNA22, RH	c-mii	------------	------------	------------	c-mii, miR, RNA22
**CA-miR414**	RH	miR, psRT, RNA22, RH	RH	RH	------------	RH	RNA22, RH	RH
**CA-miR395x**	------------	RNA22, RH	miR, psRT, RH	RH	RH	miR, psRT, RH	cmii, miR, RH	------------
**CA-miR3522**	RH	miR, RH	psRT, RH	RH	RH	RH	------------	RH
**CA-miR2950**	RNA22, RH	miR, RNA22, RH	miR, psRT, RNA22, RH	miR, psRT, RNA22, RH	miR, RNA22, RH	miR, , RH	miR, RH	miR, RNA22, RH
**CA-miR2673b**	------------	RNA22, RH	c-mii, miR, psRT, RNA22, RH	miR, psRT, RNA22, RH	RH	miR, RNA22, RH	miR, RNA22, RH	miR, RNA22, RH
**CA-miR2673a**	------------	RNA22, RH	miR, psRT, RNA22, RH	miR, psRT, RNA22, RH	RH	miR, RNA22, RH	miR, RNA22, RH	miR, RNA22, RH
**CA-miR2657b**	RH	RNA22, RH	c-mii, miR, RH	RH	------------	------------	miR, RH	------------
**CA-miR2657a**	RH	RNA22, RH	miR, RH	RH	------------	------------	miR, RH	------------
**CA-miR2108b**	psRT	psRT, RH	miR, psRT, RH	miR, RH	miR, psRT	RH	miR	------------
**CA-miR1535**	RNA22, RH	miR, RNA22, RH	miR, psRT, RNA22, RH	miR, psRT, RNA22, RH	miR, psRT, RNA22	RH	RNA22, RH	RH
**CA-miR1533**	------------	miR	miR	------------	miR, psRT	------------	cmii	------------
**CA-miR1134**	psRT	miR, psRT	miR, psRT	------------	------------	------------	miR	miR
**CA-miR1128**	RH	RH	RH	RH	RH	RH	RH	RH
**CA-miR1127**	RH	RH	RH	RH	RH	RH	RH	RH

RH, RNAhybrid; miR, miRanda; psRT, psRNATarget.

### CA-miRNA target prediction at virion-sense strand

#### ORF AV1 encoding a coat protein

The ORF AV1 encodes the coat protein (CP) on the virion-sense of DNA-A (Kallender et al., 1988). The CP gene is the most conserved area in begomoviruses (**Supplementary Figure 2A**). Since all 22 members target the AV1 gene using all algorithms, we only discussed the top five miRNAs (**Figure 5F**, **Table 3**). The CA-miR5658 in the cmii tool only targeted ToLCNDV_RE_RVA_AV1, thirteen miRNAs in all, targeting the ten isolates in miRanda (**Figures 5A, B**, **Table 4**). Furthermore, in miRanda, ChiLCV_GKP_RVA_AV1, ChiLCV_KLD_01_RVA_AV1, ChiLCV_MZP_RVA_AV1, and ChiLCV_VAR_RVA_AV1 were the targets of CA-miR838; in addition, ChiLCV_GKP/IN/21, ToYLCV_DEO_RVA/IN/23, ToLCNDV_RE_RVA_AV1, and ChiLCINV_GZB_RVA_AV1 were shows the affinities for CA-miR5658 (**Figure 5B**, **Table 4**). The begomovirus isolates ToYLCV_DEO_RVA_AV1, ChiLCV_KLD_01_RVA_AV1, ToLCNDV_RE_RVA_AV1, CLCuMuV_R_RVA_AV1, and ChiLCINV_GZB_RVA_AV1 were individually targeted by CA-miR5658 in psRNATarget (**Figure 5D**, **Table 4**). CA-miR5658 can cleave ToYLCV_DEO_RVA_AV1, ChiLCV_KLD_01_RVA_AV1, ToLCNDV_RE_RVA_AV1, CLCuMuV_R_RVA_AV1, and ChiLCINV_GZB_RVA_AV1 in RNA hybrid algorithms. The isolate ChiLCV_KLD_01:AV1 was the target of both CA-miR2673 (a and b) in RNA22 (**Figure 5E**, **Table 4**).

**Table 4:** List of top five miRNA targeting six ORFs of DNA-A, alphasatellite, and betasatellite of present diverse begomovirus isolates along with the range of MFE and expectation score obtained for miRNA through every algorithm.

	Isolates	Predicted miRNA				
		CA-miR838	CA-miR5021	CA-miR5658	CA-miR2673a	CA-miR2673b
**Predicted Targets in ORFs of different isolates**	**ChiLCV_GKP/IN/21** **ChiLCB_GKP/IN/21**	AC1*@^, AC4@^ ,betaC1^$@^	AC1*#$@, AV1#@,AC3@	AV1^#^, AC3^#$@^^, AC2^#^^, AC1^@^^	AC1#@^, AC4#@^, betaC1^#@^	AC1#@^, AC4#@^, betaC1^#@^
	**ChiLCV_GKP_RVA/IN/23** **ChLCuB_GKP_RVA/IN/23** **ChiLCAA_GKP_RVA/IN/23**	AC1^*^@^, AV1^#@^, AC2^^^, AC4^@^^, Rep^@^	AC1^*#$@^^, Rep*^#^	AC3^#$@^^, AC2^#@^^, AC1^@^^	AV1^@^, AC1^*#@^^, AC2^#@^, AC4^#@^^, betaC1^#@,^ Rep^#$^^	AV1^@^, AC1^*#@^^, AC2^#@^, AC4^#@^^, betaC1^#@^, Rep^#$^^
	**ToYLCV_DEO_RVA/IN/23** **ChLCuB_DEO_RVA/IN/23**	AC1^#@^, AC2^@^, AC3^@^, AC4^@^	AC1^*^#$^@^	AV1^#$@^, AC1^#^	AC1^#@^, AC2^@^, AC3^@^, AC4^@^	AC1^#@^, AC2^@^, AC3^@^, AC4^@^
	**ChiLCV_KLD_01_RVA/IN/23** **ChLCuB_KLD_01_RVA/IN/23**	AC1^*^, AV1^#^, betaC1^@^	AC1^#^	AV1^@$^, AC3^@^^, AC2^@^	AV1^^^, betaC1^#@^	AV1^^^, betaC1^#@^
	**ChiLCV_KLD_RVA/IN/23** **OLCuB_KLD_RVA/IN/23**	AV1^#^, AC1^$@^, AC2^$^	AC1*^#$^@	AC1^@^, AV1^@^, betaC1^#^	AC1^#$@^, AC2^$@^	AC1^#$@^, AC2^$@^
	**ChiLCV_MZP_RVA** **CLCuB_MZP_RVA/IN/23**	AC1^*^@^, AV1^#@^, AC2^^^, AC4^@^^, betaC1^$@^	AC1^*#$@^^	AC3^#$@^^, AC2^#@^^, AC1^@^^, betaC1^@^	AV1^@^, AC1^*#@^^, AC2^#@^, AC4^#@^^, betaC1^#@^^	AV1^@^, AC1^*#@^^, AC2^#@^, AC4^#@^^, betaC1^#@^^
	**ChiLCV_VAR_RVA/IN/23** **ChLCuB_VAR_RVA/IN/23**	AC1^*@^^, AV1^@#^, AC4^@^, betaC1^$^	AC1*^#$^@^	AC3^#$@^^, AC2^#@^^, AC1^@^^, betaC1^@^	AV1^@^, AC1^#@^^, AC4^@^, betaC1^#^	AV1^@^, AC1^#@^^, AC4^@^, betaC1^#@^
	**ToLCNDV_RE_RVA/IN/23** **ToLCB_RE_RVA/IN/23**	AC1^#$^@^, AC2^#$@^, AV1^@^, betaC1^#@^	AC1^*^, AC2^*^	AV1^*#$@^, AC3^#^, AC2^#^, AC1^$@^, AC4^$^	AV1^@^, AC1^#$@^^, AC2^#@$^^	AC1^#$@^^, AC1^#$@^^, AC2^#@$^^
	**CLCuMuV_R_RVA/IN/23** **ToLCB_R_RVA/IN/23**	AC1*^$^^@, AC2^@^, AV1^#@^, AC4^@^^, betaC1^#$^	AC1*^#$^@^	AC3^#^, AC2^#^, AV1^@$^	AC1^*#@^^, AC2^#@^^, AC4^#@^^	AC1^*#@^^, AC2^#@^^, AC4^#@^^
	**ChiLCINV_GZB_RVA/IN/23** **CLCuB_GZB_RVA/IN/23**	AC1*^#^@, AC4^@^	AC1*^#$^@	AV2^#^, AV1^#$@^, AC3^$^, AC1^^^, betaC1^@^	AC1^#@^^, AC4^#@^^, betaC1^#^	AC1^#@^^, AC4^#@^^, betaC1^#^
**Range of MFE (kcal/mol)& Expectation score**	**C-mii**	-14.2 to -24.8	-15 to -25.6	-14	-23.2	-23.2
	**miRanda**	-10.67 to -23.94	-9.8 to -24.21	-10.94 to -17.92	-9.18to -26.17	-9.18to -26.17
	**psRNATarget (Expectation)**	3 to 7	3.5 to 6	4.5 to 7	3 to 6	3 to 6
	**RNA22**	-12 to -15.5	-16.2	-12.6 to -16.2	-13.3 to -23.6	-13.3 to -23.6
	**RNAhybrid**	-20.9 to -30.2	-20.1 to -22.7	-20.5 to -27.4	-20.7 to -32.2	-20.7 to -32.2

^*^ c-mii, **^#^** miRanda, **^$^** psRNATarget, **^@^** RNAHybrid, **^^^** RNA22.

#### ORF AV2 encoding a pre-coat protein

The pre-coat protein (AV2), encoded by the virion-sense strand of DNA-A, is crucial for the transport and motility of monopartite begomoviruses inside the host cell (**Supplementary Figure 2A**) (Wang et al., 2018). It is around 365 bp long and ranges from ~149 bp to 514 bp. All 20 members targeted separate loci, except for two CA-miRNAs, CA-miR837-5p and CA-miR1533 (**Figure 5F**, **Table 3**). The miRanda method would target TYLCV_DEO_RVA_AV2 and ChiLINCV_GZB_RVA_AV2 isolates at two loci (**Figure 5B**, **Table 3**). The miRanda method would target TYLCV_DEO_RVA_AV2 and ChiLINCV_GZB_RVA_AV2 isolates at two loci (**Figure 5B**, **Table 3**). The psRNATarget algorithms targeted eight begomovirus AV2 sequences, excluding ChiLCV_KLD_01_RVA_AV2 and ChiLCINV_GZB_RVA/IN/23 (**Table 4**). Furthermore, CA-miR1134 and CA-miR5041 in psRNATarget methods have an affinity for CLCuMuV_R_RVA_AV2 and ChiLCV_Gkp_AV2. In RNA22, CA-miR2950, CA-miR1535, and CA-miR5015b all targeted ChiLCV_Gkp_AV2 (**Figure 5D**, **Table 3**). Among the top 5 miRNAs, CA-miR5658 binds the ChiLCINV_GZB_RVA_AV2 in RNAhybrid and is part of a 15-miRNA family that targets the AV2 genes of 7 isolates (**Figure 5C**, **Table 4**).

### CA-miRNA target prediction at complementary-sense strand

#### ORF AC1 encoding a replication initiator protein

The sole viral protein essential for replication is the multifunctional oligomeric protein known as the replication initiator protein (Rep) (**Supplementary Figure 2A**) (Elmer et al., 1998). Each of the 22 miRNA members targets the AC1 ORF (**Figure 5F**, **Table 3**). Except for isolate ChiLCV_KLD_01_RVA_AC1, CA-miR5021 in C-mii targets nine different begomovirus isolates. With the same exception, CA-miR2673 (a and b) exhibited binding and cleavage affinity for all the begomovirus isolates in miRanda and RNAhybrid (**Figures 5B, C**, **Table 4**). Similarly, excluding ChiLCV_KLD_01_RVA_AC1, all isolates in the psRNATarget algorithms exhibit interaction with CA-miR5021 (**Figure 5D**, **Table 4**). Furthermore, apart from ToYLCV_DEO_RVA_AC1, ChiLCV_KLD_RVA_AC1, and ChiLCV_KLD_RVA_AC1, miR2673 (a and b) also targeted the seven isolates in RN22. The most targeted ORF out of the six in all five algorithms is ORF AC1 (**Figure 5F**, **Table 3**; **Supplementary Table 1**).

#### ORF AC2 encoding a transcription activator protein

Virion-sense genes from DNA-A and DNA-B get activated by the TrAP protein (**Supplementary Figure 2A**) (Pandey et al., 2009; Gopel et al., 2007; Trinks et al., 2005; Wang et al., 2003) present on the complementary-sense strand. All candidate miRNA members, except CA-miR1533 and CA-miR1134, target ORF AC2 (**Figure 5F**, **Table 3**). ToLCNDV_RE_RVA_AC2 was the target of CA-miR5021 in C-mii. Additionally, using miRanda algorithms we found CA-miR5658 interacted with the ChiLCV isolates GKP_AC2, GKP_RVA_AC2, MZP_RVA_AC2, and VAR_RVA_AC2, along with ToLCNDV_RE_RVA_AC2 and CLCuMuV_R_RVA_AC2 (**Figure 5B**, **Table 4**). The begomovirus isolates ChiLCV_KLD_RVA_AC2 and ToLCNDV_RE_RVA_AC2 exhibited cleavage contacts with CA-miR838 and miR2673 (a and b) in psRNATarget, respectively (**Figure 5D**, **Table 4**). RNAhybrid algorithms identify miR2673 (a and b) as the topmost, targeting six isolates (**Figure 5C**, **Table 4**). Furthermore, CA-miR5658 had the highest affinity for four ChiLCV isolates: GKP_AC2, GKP_RVA_AC2, MZP_RVA_AC2, and VAR_RVA_AC2 in RNA22.

#### ORF AC3 encoding a replication enhancer protein

The replication enhancer protein, or REn, promotes the accumulation of the viral DNA within the host. The role of the Rep/REn association in viral DNA replication has been reported (**Supplementary Figure 2A**) (Gutierrez, 2002). We observed that 20 miRNA members were targeting the AC3 gene in our *in-silico* target identification process (**Figure 5F**, **Table 3**). We seemed to have no targets for AC3 in C-mii (**Figure 5A**, **Table 4**). Furthermore, the ChiLCV isolates GKP_AC3, GKP_RVA_AC3, MZP_RVA_AC3, and VAR_RVA_AC3 were the most effective targets for CA-miR5658 in the miRanda, psRNATarget, RNAhybrid, and RNA22 algorithms (**Figures 5B–E**, **Table 4**).

#### ORF AC4

The Rep protein contains ORF AC4; however, it is in a distinct orientation, so it encodes for an alternate protein (**Supplementary Figure 2A**). Infected tissues had systemic necrosis when the Rep and AC4 proteins were co-expressed (van et al., 2002). Nineteen miRNAs were predicted to target this gene (**Figure 5F**, **Table 3**). Except for C-mii, all other algorithms show favourable results for AC4 with CA-miR2673 (a and b) (**Figure 5A**). These algorithms target ChiLCV isolates GKP_AC4, GKP_RVA_AC4, and MZP_RVA_AC4 for miRanda, psRNATarget, RNAhybrid, and RNA22 (**Figures 5B, D, E**, **Table 4**). Furthermore, CA-miR838 cleavage association with ChiLCV_GKP/IN/21, ToYLCV_DEO_RVA/IN/23, ChiLCV_VAR_RVA/IN/23, and CLCuMuV_R_RVA/IN/23 was also identified by RNAhybrid (**Figure 5C**, **Table 4**).

#### Associated satellite targeted by predicted CA-miRNA

Begomoviruses include two forms of circular DNA satellites: alphasatellites and betasatellites (Fiallo-Olivé et al., 2021; Gnanasekaran et al., 2019; Zhou, 2013). Alphasatellites encode parts of nanoviruses that code for replication-initiation protein (Rep). BetaC1, a single betasatellite ORF, is associated with numerous monopartite begomoviruses that trigger the emergence of classical disease symptoms (**Supplementary Figure 2B**) (Gnanasekaran et al., 2019; Li et al., 2018). In all, 16 and 19 miRNA-predicted members targeted the alphasatellites and betasatellites, respectively (**Table 3**). We identified that ChiLCAA_GKP_RVA_rep only interacted with CA-miR5021 using the C-mii tool. Furthermore, in miRanda and psRNATarget, miR2673 (a and b) had an affinity for ChiLCAA_GKP_RVA_rep (**Table 4**). In contrast, CA-miR838 specifically targeted it in RNA hybrids. MiR2673 (a and b) preferentially targeted the ChiLCB_GKP_betaC1, ChLCuB_GKP_RVA_betaC1,ChLCuB_KLD_01_RVA_betaC1,CLCuB_MZP_RVA_betaC1, and ChLCuB_VAR_RVA_betaC1 in both miRanda and RNAhybrid (**Table 4**). According to the psRNATarget targeting results, CA-miR838 has cleavage binding affinity for ToLCB_R_RVA_betaC1, CLCuB_MZP_RVA_betaC1, and ChiLCB_GKP_betaC1 (**Table 4**). The evaluations of RNA hybrid algorithms predict the greatest number of targets among all begomovirus satellites (**Table 4**).

#### Mapping of miRNA-begomovirus interaction

**Figure 7:**
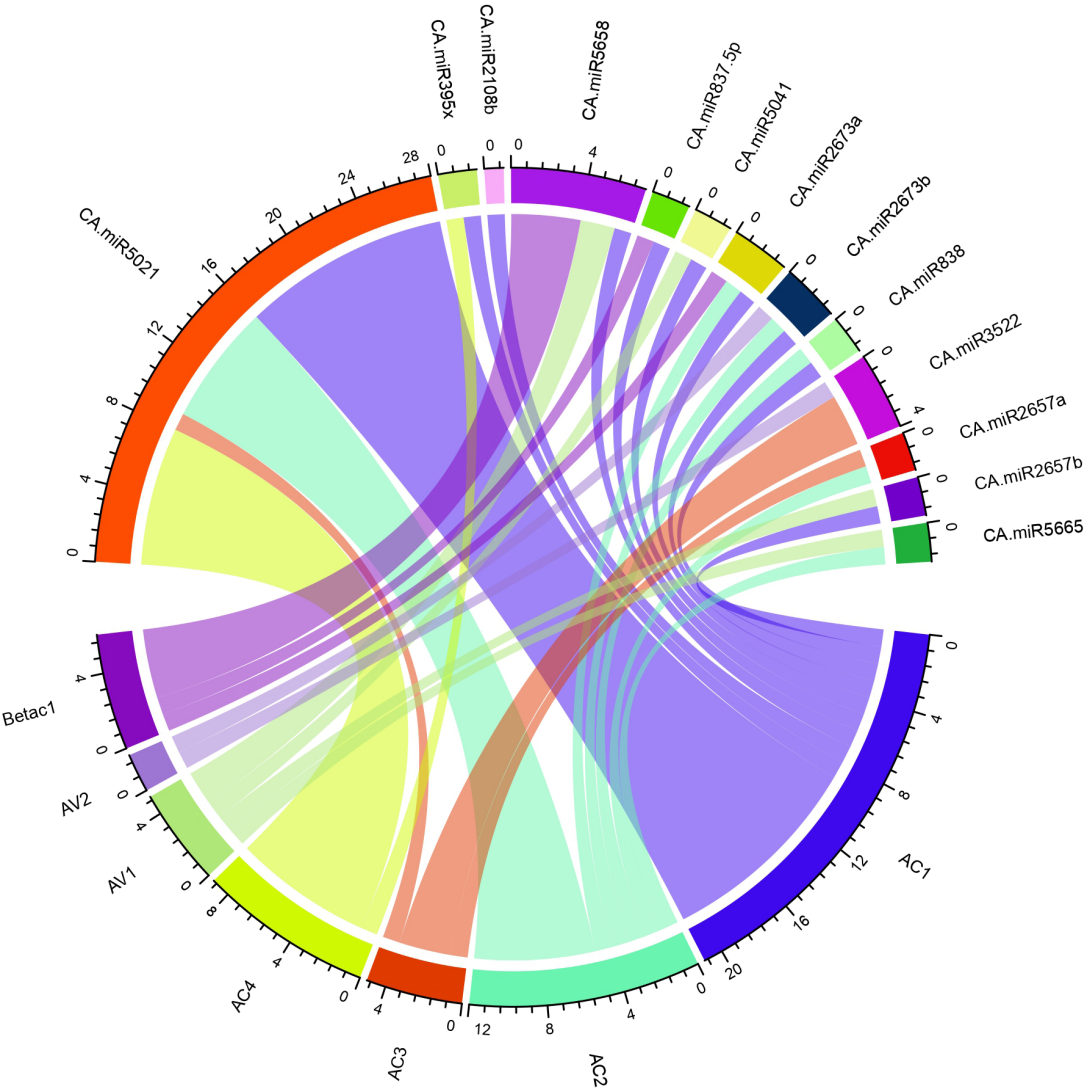
The begomovirus genome’s schematic interaction diagram for the chilli-target interaction is shown. The chilli miRNAs predicted by the algorithm cmii in the genome of begomovirus are summarised in a circular plot (Circos) generated through R-program. The outer ring denotes the genetic components of the begomovirus (ORFs) and predicted chilli miRNAs. The coloured lines represent the interaction between begomovirus and target ORFs.

To precisely combine the biologically reliable data for the miRNA-host gene connection analysis, we created circos plots through the R- program for the visualisation of the miRNA target (**Table 4**). The begomovirus genome shows the mapped chilli miRNAs (**Figure 7**). We evaluated chilli miRNAs and their begomovirus ORF targets, as identified by at least four of the algorithms employed in this study, to ensure the most effective visualisation and clarity for better readability.

#### Thermodynamic stability: free energy estimation for miRNA–mRNA heterodimer

The free energy (ΔG) of miRNA-mRNA duplex for those miRNAs that were supported by all five predicted tools was evaluated. The RNAcofold algorithm’s free energy (ΔG) estimation was based on the alignment (miRNA-mRNA) result of psRNATarget. Six duplexes were identified, with the lowest free energy (ΔG) of > -20 kcal/mol for miR838 and miR2673 (a and b) (**Table 5**). The miRNA-mRNA complex is thought to be highly thermodynamically stable, and the miRNA-mRNA association is stronger whenever the free energy is low. This constitutes essential information because it increases the likelihood that stable miRNA-mRNA binding will be recognized as an actual interaction (Riolo et al., 2020).

**Table 5:** Heterodimer free energy (ΔG) of top five predicted miRNA along with binding range and alignment of miRNA: Target duplex.

miRNA	Target Acronyms	miRNA length	Target length	CA-miRNA aligned fragment	Alignment	Begomovirus aligned fragment	ΔG (Kcal/ mol) Heterodimer binding
CA-miR838	ToLCNDV_RE_RVA_AC2	21	20	UCUUCUUCUUCUUCUUCUUCA	:::::::::::::::. :	CCCAGAAGAAGAAGAAGAGCA	-23.62
	ToLCNDV_RE_RVA_AC1	21	20	UCUUCUUCUUCUUCUUCUUCA	:::::::::::::::. :	CCCAGAAGAAGAAGAAGAGCA	-23.62
	ChiLCV_KLD_RVA_AC1	21	20	UCUUCUUCUUCUUCUUCUUCA	::. :::::.:..:: :.::	UGGCGAAGAGGGGGACCAGGA	-16.35
	ChiLCV_KLD_RVA_AC2	21	20	UCUUCUUCUUCUUCUUCUUCA	::. :::::.:..:: :.::	UGGCGAAGAGGGGGACCAGGA	-16.35
	CLCuMuV_R_RVA_AC1	21	19	UCUUCUUCUUCUUCUUCUUCA	::: ::.::: ::::: :	CCUAGACGAGGAA-AAGAACA	-10.58
	ChiLCB_GKP/IN/21/betaC1	21	20	UCUUCUUCUUCUUCUUCUUCA	:: .:::::..:: :.::	AUAACGAGAAGGGGAUGGAGU	-16.87
	ToLCB_R_RVA/IN/23/betaC1	21	19	UCUUCUUCUUCUUCUUCUUCA	::::: ::::: :.:::	ACAAGAACAAGAA-AGGAAUG	-11.05
	ChLCuB_VAR_RVA/IN/23/betaC1	21	20	UCUUCUUCUUCUUCUUCUUCA	:: :: .:::::..:: :.	AUAAUAACGAGAAGGGGAUGG	-13.54
CA-miR5658	ToLCNDV_RE_RVA_AV1	21	20	AUGAUGAUGAUUAUGGUGAUC	::.:.:.:::.:.::::	AUGCAUCGUGAUCGUUAUCAA	-19.50
	ChiLCV_VAR_RVA_AC3	21	20	AUGAUGAUGAUUAUGGUGAUC	:..:..::::.: .::::	UUUUGCUGUAAUUAGUAUCAA	-10.01
	ChiLCV_GKP_RVA_AC3	21	20	AUGAUGAUGAUUAUGGUGAUC	:..:..::::.: .::::	UUUUGCUGUAAUUAGUAUCAA	-10.01
	ToLCNDV_RE_RVA_AC1	21	20	AUGAUGAUGAUUAUGGUGAUC	:.:.: ::::: :.::::	AUUUAUCUAAAUCAGCGUCAU	-11.88
	ToLCNDV_RE_RVA_AC4	21	20	AUGAUGAUGAUUAUGGUGAUC	:.:.: ::::: :.::::	AUUUAUCUAAAUCAGCGUCAU	-11.88
	ChiLCV_Gkp_AC3	21	20	AUGAUGAUGAUUAUGGUGAUC	:..:..::::.: .::.:	UUUUGCUGUAAUUAGUAUUAA	-7.82
	ChiLINCV_GZB_RVA_AV1	21	20	AUGAUGAUGAUUAUGGUGAUC	::.:.:::.::.:: :	CUUGUCCGUGAUCGUCGUCCU	-17.93
	ChiLCV_KLD_01_RVA_AV1	21	20	AUGAUGAUGAUUAUGGUGAUC	: :::.:.:.:: . .::::	GUUCAUCGUGAUAGGUAUCAG	-12.45
	CLCuMuV_R_RVA_AV1	21	20	AUGAUGAUGAUUAUGGUGAUC	: :::.:.:.:: . .::::	GUUCAUCGUGAUAGGUAUCAA	-12.45
	ChiLCV_Gkp_AC3	21	20	AUGAUGAUGAUUAUGGUGAUC	: .:::::: :.::.:	AUAAAAUAUAAUCUUUAUUAA	-7.57
	ChiLINCV_GZB_RVA_AV1	21	20	AUGAUGAUGAUUAUGGUGAUC	::. . .:::.:.::::	AUGCAUAGAGAUCGUUAUCAG	-12.14
	TYLCV_DEO_RVA_AV1	21	20	AUGAUGAUGAUUAUGGUGAUC	:::: :. .:::.:..:::	GAUCUGCGGGAUCGUUGUCAG	-10.25
	ChiLINCV_GZB_RVA_AC3	21	20	AUGAUGAUGAUUAUGGUGAUC	:..:..::::.: .:: :	UUUUGCUGUAAUUAGUAUAAA	-8.49
	ChiLCV_MZP_RVA_AC3	21	20	AUGAUGAUGAUUAUGGUGAUC	:..:..::::.: .:: :	UUUUGCUGUAAUUAGUAUAAA	-5.64
	CLCuB_MZP_RVA/betaC1	21	20	AUGAUGAUGAUUAUGGUGAUC	::: :: :.::: ::::.	UAUCCACACAGUCACCAUCGC	-11.59
CA-miR5021	ChiLINCV_GZB_RVA_AC1	20	19	UAAGAAGAGGAAGAUCAAAA	::::: :::::.::::.: :	UUUUGUUCUUCUUCUUUUGA	-18.09
	TYLCV_DEO_RVA_AC1	20	19	UAAGAAGAGGAAGAUCAAAA	:::: ::::::.::::.: :	UUUUUAUCUUCUUCUUUUAA	-16.47
	ChiLCV_MZP_RVA_AC1	20	19	UAAGAAGAGGAAGAUCAAAA	:::: :::::.::::.: :	UUUUCUUCUUCUUCUUUUGA	-17.39
	ChiLCV_VAR_RVA_AC1	20	19	UAAGAAGAGGAAGAUCAAAA	:::: :::::.::::.: :	UUUUCUUCUUCUUCUUUUGA	-17.39
	ChiLCV_GKP_RVA_AC1	20	19	UAAGAAGAGGAAGAUCAAAA	:::: :::::.::::.: :	UUUUCUUCUUCUUCUUUUGA	-17.39
	ChiLCV_KLD_RVA_AC1	20	19	UAAGAAGAGGAAGAUCAAAA	::: ::::::.::::.: :	UUUCUAUCUUCUUCUUUUGA	-15.58
	CLCuMuV_R_RVA_AC1	20	17	UAAGAAGAGGAAGAUCAAAA	:::: :::::.:::::::	UUUU--UCUUCUUCUUCUUU	-17.77
	ChiLCV_Gkp_AC1	20	19	UAAGAAGAGGAAGAUCAAAA	::::: :::::.::::.	UUUUGUUCUUCUUCUUUCGA	-17.24
CA-miR2673a	ToLCNDV_RE_RVA_AC2	22	21	CCUCUUCUUCUUCUUCUUCUUC	::::::::::::::::	CCCCCAGAAGAAGAAGAAGAGC	-25.05
	ToLCNDV_RE_RVA_AC1	22	21	CCUCUUCUUCUUCUUCUUCUUC	::::::::::::::::	CCCCCAGAAGAAGAAGAAGAGC	-25.05
	ChiLCV_KLD_RVA_AC1	22	21	CCUCUUCUUCUUCUUCUUCUUC	:. :::::.:..:: :.:::	GGCGAAGAGGGGGACCAGGAGU	-18.45
	ChiLCV_KLD_RVA_AC2	22	21	CCUCUUCUUCUUCUUCUUCUUC	:. :::::.:..:: :.:::	GGCGAAGAGGGGGACCAGGAGU	-18.45
	ChiLCAA_GKP_RVA/IN/23	22	21	CCUCUUCUUCUUCUUCUUCUUC	:.. :::::::.:::	CCUGUUGGGCAAGAAGAGGAGU	-13.65
CA-miR2673b	ToLCNDV_RE_RVA_AC2	22	21	CCUCUUCUUCUUCUUCUUCUUC	::::::::::::::::	CCCCCAGAAGAAGAAGAAGAGC	-25.05
	ToLCNDV_RE_RVA_AC1	22	21	CCUCUUCUUCUUCUUCUUCUUC	::::::::::::::::	CCCCCAGAAGAAGAAGAAGAGC	-25.05
	ChiLCV_KLD_RVA_AC1	22	21	CCUCUUCUUCUUCUUCUUCUUC	:. :::::.:..:: :.:::	GGCGAAGAGGGGGACCAGGAGU	-18.45
	ChiLCV_KLD_RVA_AC2	22	21	CCUCUUCUUCUUCUUCUUCUUC	:. :::::.:..:: :.:::	GGCGAAGAGGGGGACCAGGAGU	-18.45
	ChiLCAA_GKP_RVA/IN/23	22	21	CCUCUUCUUCUUCUUCUUCUUC	:.. :::::::.:::	CCUGUUGGGCAAGAAGAGGAGU	-13.65

## Discussion

Both animal and plant cells use miRNAs (miRNAs) as post-transcriptional regulators of gene expression. miRNAs regulate gene expression by binding to miRNA-responsive elements (mREs) on target mRNAs, causing significant changes in a variety of physiological and pathological processes (Bhaskaran and Mohan, 2014; Chen, 2005; O’Brien et al., 2018; Shang et al., 2023). Understanding the miRNA regulatory network requires finding miRNA-mRNA target interactions. Using amiRNA-based technology, host-derived miRNAs were exploited to silence the genome of plant-infecting viruses (Niu et al., 2006; Petchthai et al., 2018). To combat plant viruses, miRNA has lately been utilised to genetically alter crops as a new endogenous domain for gene control (Sharma et al., 2022; Tang and Chu, 2017). The primary strategies for understanding miRNA targets were presented in the current work. We used the C-mii algorithm to predict 20 miRNA families from chilli transcriptome SRA data. During the analysis of projected miRNA, we discovered that family CA-miR5021 had 47.25% GC content in their pre-miRNA sequence, with AU content ranging from 42.75 to 80.56 (**Table 2**). The detection of a significant increase in the GC content of stress-regulated miRNA sequences further supports miRNAs’ role as ubiquitous regulators under stressful conditions. In plants such as *Arabidopsis thaliana*, GC content may be considered an important indicator for predicting stress-induced miRNAs (Mishra et al., 2009). Uracil was shown to be prominent in the first position of the mature miRNA sequence, indicating its role in miRNA-mediated plant regulation (Luo et al., 2013; Dhandapani et al., 2011; Unver et al., 2010; Zhang et al., 2008). In the case of pre-miRNA, adenine, and uracil appear to be dominating, which is consistent with prior findings in *Brassica rapa L.* and *Gossypium arboretum L*. Previous studies have shown that U at the first position in a sequence plays an important role in miRNA-mediated regulation in plants (Dhandapani et al., 2011; Unver et al., 2010; Zhang et al., 2008). Predictive computation before laboratory verification for these miRNA-mRNA interactions is typically the best strategy for accomplishing disease management objectives. There were various computer analytical methods, including C-mii, RNAhybrid, RNA22, psRNATarget, andmiRanda, each with their own method of predicting miRNA targets (**Table 1**). In the current work, mature chilli twenty miRNA families were chosen to generate a begomovirus-resistant chilli cultivar, as well as their interactions with begomovirus AC1, AC2, AC3, AC4, AV1, AV1, beta C1, and rep ORFs (**Figure 5A–E**, **Table 4**). We focused mainly on the CA-miRNA predicted by all five algorithms (CA-miR838, CA-miR5658, CA-miR5021, CA-miR2673a, and CA-miR2673b) (**Table 4**). The consensus hybridization sites for CA-miR838 (locus 2052), CA-miR5658 (locus 817-819), and CA-miR5021 (locus 1542) were found (**Figure 6**). CA-miR2673 (a and b), observed among the ORFs of begomovirus isolates, has the maximum number of targets and >15 locus contacts in three algorithms, as well as the highest affinity for all satellites and ORFs. MFE is an important component in tracking site accessibility for precisely identifying a secondary duplex structure, which is required to assess the thermodynamic sustainability of the miRNA-mRNA duplex (Doench and Sharp, 2004; Peterson et al., 2014). Strong hybridization interactions between miRNA and mRNA result in prolonged stability of the RNA duplex (**Figure 2**). The CA-miR2673 (a and b) interacting with ORFs in the RNAhybrid and psRNATarget had the lowest MFE range (-20.7 to -32.2 kcal/mol) and expectation score (3 to 6) (**Table 4**). A higher association between the miRNA-target duplex suggested a lower expectation value (Dai and Zhao, 2011). Since it can be costly and laborious to determine miRNA-mRNA interactions through experimentation, precise algorithmic prediction of miRNA targets is of utmost importance. To prevent false-positive outcomes in this investigation, we created three strategies at the individual, union, and intersection levels. Several *in silico* algorithms are combined in the union approach to estimate if a target is true or false. By lowering specificity, this method raised the sensitivity level of identified targets. As compared to this method, the intersectional level of research relies solely on the intersection of two or more computational algorithms, which lowers sensitivity and increases the specificity of anticipated targets. As illustrated in **Figure 6**, **Supplementary Figure 3**, our results revealed that we had succeeded in predicting and estimating unique targets with good performance utilising both algorithmic approaches. This has contributed to the discovery of highly promising predicted miRNA targets for plant viruses by genome-wide characterization and thorough investigation (Wenzhi et al., 2024; Ashraf et al., 2021; Gaafar and Ziebell, 2020; Iqbal et al., 2017). To prevent begomovirus infection in chilli cultivars, the current study was designed using an equally innovative computational approach for suggesting novel targets against begomovirus. A biological system’s stability is assessed using free energy. Even though a miRNA-mRNA complex has lower free energy, breaking its bond requires more energy. The duplexes with the lowest free energy (ΔG) of more than -20 kcal/mol were with miR838 and miR2673 (a and b) (**Table 5**). The best method to reduce productivity and quality reductions is to generate varietal-resistant chillies to combat viral infection. Because begomoviruses have such a high degree of genetic variability, determining the favourable agronomic features of resistant hosts is challenging. The potential miRNAs (CA-miR838, CA-miR5658, CA-miR5021, CA-miR2673a, and CA-miR2673b) and the chilli callus’s great capacity to regenerate can be used to create ChiLCD-tolerant chilli cultivars. Our findings revealed that, according to psRNATarget, all of the examined outcomes have been associated with post-transcriptional regulation by cleavage or complete inhibition. RNA interference (RNAi) technology has been widely used to identify novel cellular activities and test host-delivered factors against viruses (Agrawal et al., 2003; Kurreck, 2009). We isolated mature chilli miRNAs that have been computationally confirmed and identified as genomic targets for the begomovirus. To the best of our knowledge, this was the first time that miRNA was predicted from chilli transcriptome data that had not previously been reported in miRbase. Additional laboratory research will be necessary to validate these findings, which might have a significant impact on the management of begomoviruses that infect chilli crops.

## Conclusion

The begomovirus, which infects chilli crops worldwide, is the leading cause of the continuing ChiLCD epidemic, reducing yield in all cultivars grown globally. This study used computer-based methods to find the sites in the begomovirus genome where mature miRNAs from chillies can bind. Of the 22 predicted CA-miRNAs, CA-miR838, CA-miR5658, CA-miR5021, and CA-miR2673 (a and b) were the ones that interacted with begomovirus genomes and were validated by all five algorithms. Strong complementarities between these miRNAs and the AC1 (Rep) and AC2 (TrAP) genes were also observed. Based on the consensus of multiple approaches used, only two potential CA-miRNAs (CA-miR838 and CA-miR5658) were found to be the most efficient for targeting the begomovirus genome (targeted loci at 2052 and 817-819, respectively). Thus, determining the crucial targets of these two miRNAs associated with begomovirus genome silencing and understanding their role in a genomic-editing-based transformation approach would be a future challenge. Using chilli transformation techniques, predicted novel targets can be designed for the production of begomovirus-resistant chilli cultivars.

## Data Availability

The datasets presented in this study can be found in online repositories. The names of the repository/repositories and accession number(s) can be found in the article/**Supplementary Material**.

## Supplementary material

Supplementary Figure 1 A computational methodology for predicting miRNAs offered by hosts using the chilli transcriptome SRA data. Diagram illustrating the many stages of prediction used to examine miRNA targets encoded by chilli found in the genome of begomoviruses. The biological data includes SRA data on chilli, ten begomoviruses.

Supplementary Figure 2 Genome organization of begomovirus **(A)** DNA-A **(B)** Betasatellite, **(C)** Alphasatellite

Supplementary Figure 3 Diagrammatic representation of 22 predicted miRNA, its Targets within begomovirus genome and total algorithms validated each predicted miRNA.
