# Supplementary material for: Predicting candidate miRNAs for targeting begomovirus to induce sequence-specific gene silencing in chilli plants
Source: Front Plant Sci. 2024 Sep 23;15:1460540. doi: 10.3389/fpls.2024.1460540 (PMC11456425; doi:10.3389/fpls.2024.1460540)
Supplement: Supplementary file 1 [file Table1.docx]

| **Accession No.** | **Identified Begomovirus** | **Genome** | **Isolate** | **Acronyms** | **Genome size** |
| --- | --- | --- | --- | --- | --- |
| **MZ540908** | Chilli Leaf Curl Virus | DNA-A | GKP | **ChiLCV_GKP/IN/21** | **2967 bp** |
| **MZ540909** | Chili Leaf Curl Betasatellite | Betasatellite | GKP | **ChiLCB_GKP/IN/21** | **1389 bp** |
| **OQ107574** | Chilli Leaf Curl Virus | DNA-A | GKP_RVA | **ChiLCV_GKP_RVA/IN/23** | **2767 bp** |
| **OQ107575** | Chili Leaf Curl Betasatellite | Betasatellite | GKP_RVA | **ChLCuB_GKP_RVA/IN/23** | **1390 bp** |
| **OQ091758** | Tomato Yellow Leaf Curl Virus | DNA-A | DEO_RVA | **ToYLCV_DEO_RVA/IN/23** | **2792 bp** |
| **OQ091759** | Chilli Leaf Curl Betasatellite | Betasatellite | DEO_RVA | **ChLCuB_DEO_RVA/IN/23** | **1390 bp** |
| **OQ161704** | Chilli Leaf Curl Virus | DNA-A | KLD_01_RVA | **ChiLCV_KLD_01_RVA/IN/23** | **2758 bp** |
| **OQ134776** | Chili Leaf Curl Betasatellite | Betasatellite | KLD_01_RVA | **ChLCuB_KLD_01_RVA/IN/23** | **1369 bp** |
| **OQ148478** | Chilli Leaf Curl Virus | DNA-A | KLD_RVA | **ChiLCV_KLD_RVA/IN/23** | **2764 bp** |
| **OQ716463** | Okra Leaf Curl Betasatellite | Betasatellite | KLD_RVA | **OLCuB_KLD_RVA/IN/23** | **1375 bp** |
| **OQ440388** | Chilli Leaf Curl Virus | DNA-A | MZP_RVA | **ChiLCV_MZP_RVA** | **2766 bp** |
| **OQ440389** | Cotton Leaf Curl Betasatellite | Betasatellite | MZP_RVA | **CLCuB_MZP_RVA** | **1376 bp** |
| **OQ440390** | Chilli Leaf Curl Virus | DNA-A | VAR_RVA | **ChiLCV_VAR_RVA/IN/23** | **2767 bp** |
| **OQ440391** | Chili Leaf Curl Betasatellite | Betasatellite | VAR_RVA | **ChLCuB_VAR_RVA/IN/23** | **1378 bp** |
| **OQ267623** | Tomato Leaf Curl New Delhi Virus | DNA-A | RE_RVA | **ToLCNDV_RE_RVA/IN/23** | **2766 bp** |
| **OQ267624** | Tomato Leaf Curl Betasatellite | Betasatellite | RE_RVA | **ToLCB_RE_RVA/IN/23** | **1376 bp** |
| **OQ440386** | Cotton Leaf Curl Multan Virus | DNA-A | R_RVA | **CLCuMuV_R_RVA/IN/23** | **2738 bp** |
| **OQ440387** | Tomato Leaf Curl Betasatellite | Betasatellite | R_RVA | **ToLCB_R_RVA/IN/23** | **1356 bp** |
| **OQ267621** | Chilli Leaf Curl India Virus | DNA-A | GZB_RVA | **ChiLCINV_GZB_RVA/IN/23** | **2963 bp** |
| **OQ267622** | Cotton Leaf Curl Betasatellite | Betasatellite | GZB_RVA | **CLCuB_GZB_RVA/IN/23** | **1436 bp** |

**Table S1** Details on the ten begomoviruses, ten betasatellite and one alphasatellite associated with the chilli crop that causes Chilli leaf curl Disease (ChiLCD) were used in the current study as targets

**Table S2** Chilli SRA transcriptome data quality check by QUAST tool.

| **QUAST REPORT** | |
| --- | --- |
| #Contigs {>=0bp} | 64198 |
| #Contigs {>=1000bp} | 10291 |
| Total length {>=0bp} | 37136480 |
| Total length {>=1000bp} | 18384223 |
| 3 contig | 20975 |
| Largest contig | 8521 |
| Total Length | 25896435 |
| **GC (%)** | **41.40** |
| N50 | 1464 |
| N90 | 647 |
| auN | 1733.1 |
| L50 | 5797 |
| L90 | 16431 |
| **3N’s per 100 kbp** | **0.00** |

**Table S3. List of predicted targeting sites by potential CA-miRNA within the begomovirus genome using the C-mii tool.**

| **miRNA** | **Begomovirus Isolate** | **Target** | **MFE ( Kcal/Mol)** |
| --- | --- | --- | --- |
| CA-miR5021 | ToYLCV_DEO_RVA | 712 | -16.3 |
| CA-miR5021 | ToYLCV_DEO_RVA | 712 | -15.9 |
| CA-miR837-5p | ToYLCV_DEO_RVA | 2062 | 13.7 |
| CA-miR5021 | CLCuMuV_R_RVA | 696 | -19.1 |
| CA-miR5021 | CLCuMuV_R_RVA | 693 | -19.9 |
| CA-miR5021 | CLCuMuV_R_RVA | 693 | -20.2 |
| CA-miR5021 | CLCuMuV_R_RVA | 702 | -19.8 |
| CA-miR5021 | CLCuMuV_R_RVA | 696 | -18.3 |
| CA-miR5021 | CLCuMuV_R_RVA | 696 | -22.1 |
| CA-miR2673a | CLCuMuV_R_RVA | 2021 | -23.2 |
| CA-miR837-5p | CLCuMuV_R_RVA/ | 2018 | -14.8 |
| CA-miR837-5p | CLCuMuV_R_RVA | 2018 | -15.8 |
| CA-miR838 | CLCuMuV_R_RVA | 2020 | -24.8 |
| CA-miR2673b | CLCuMuV_R_RVA | 2021 | -23.2 |
| CA-miR5021 | ChiLCV_GKP_RVA | 696 | -19.1 |
| CA-miR5021 | ChiLCV_GKP_RVA | 693 | -21 |
| CA-miR5021 | ChiLCV_GKP_RVA | 693 | -20.1 |
| CA-miR5021 | ChiLCV_GKP_RVA | 702 | -22.7 |
| CA-miR5021 | ChiLCV_GKP_RVA | 696 | -17.4 |
| CA-miR5021 | ChiLCV_GKP_RVA | 696 | -20.6 |
| CA-miR5021 | ChiLCV_GKP_RVA | 702 | -16.4 |
| CA-miR2673a | ChiLCV_GKP_RVA | 2050 | -23.2 |
| CA-miR837-5p | ChiLCV_GKP_RVA | 2047 | -14.8 |
| CA-miR837-5p | ChiLCV_GKP_RVA | 2047 | -14.4 |
| CA-miR838 | ChiLCV_GKP_RVA | 2049 | -24.8 |
| CA-miR2673B | ChiLCV_GKP_RVA | 2050 | -23.2 |
| CA-miR5021 | ChiLCINV_GZB_RVA | 699 | -16.1 |
| CA-miR5021 | ChiLCINV_GZB_RVA | 696 | -16.2 |
| CA-miR5021 | ChiLCINV_GZB_RVA | 693 | -16.1 |
| CA-miR5021 | ChiLCINV_GZB_RVA | 702 | -18.2 |
| CA-miR5021 | ChiLCINV_GZB_RVA | 696 | -16.1 |
| CA-miR5021 | ChiLCINV_GZB_RVA | 702 | -16.9 |
| CA-miR837-5p | ChiLCINV_GZB_RVA | 2043 | -12.5 |
| CA-miR837-5p | ChiLCINV_GZB_RVA | 2043 | -11.6 |
| CA-miR838 | ChiLCINV_GZB_RVA | 2048 | -20 |
| CA-miR5021 | ChiLCV_GKP/IN/21 | 692 | -15 |
| CA-miR5021 | ChiLCV_GKP/IN/22 | 689 | -15.1 |
| CA-miR5021 | ChiLCV_GKP/IN/23 | 686 | -16.1 |
| CA-miR5021 | ChiLCV_GKP/IN/24 | 689 | -17.3 |
| CA-miR5021 | ChiLCV_GKP/IN/25 | 695 | -16.1 |
| CA-miR5021 | ChiLCV_GKP/IN/26 | 2053 | -20 |
| CA-miR838 | ChiLCV_KLD_01_RVA | 2051 | -17 |
| CA-miR838 | ChiLCV_KLD_RVA | 701 | -14.2 |
| CA-miR5021 | ChiLCV_MZP_RVA | 696 | -19.1 |
| CA-miR5021 | ChiLCV_MZP_RVA | 693 | -21 |
| CA-miR5021 | ChiLCV_MZP_RVA | 693 | -20.1 |
| CA-miR5021 | ChiLCV_MZP_RVA | 702 | -22.7 |
| CA-miR5021 | ChiLCV_MZP_RVA | 696 | -17.4 |
| CA-miR5021 | ChiLCV_MZP_RVA | 696 | -20.6 |
| CA-miR5021 | ChiLCV_MZP_RVA | 702 | -16.4 |
| CA-miR2673a | ChiLCV_MZP_RVA | 2050 | -23.2 |
| CA-miR837-5p | ChiLCV_MZP_RVA | 2047 | -14.8 |
| CA-miR837-5p | ChiLCV_MZP_RVA | 2047 | -14.4 |
| CA-miR838 | ChiLCV_MZP_RVA | 2049 | -24.8 |
| CA-miR2673b | ChiLCV_MZP_RVA | 2050 | -23.2 |
| CA-miR5021 | ChiLCV_VAR_RVA | 696 | -19.1 |
| CA-miR5021 | ChiLCV_VAR_RVA | 693 | -21 |
| CA-miR5021 | ChiLCV_VAR_RVA | 693 | -20.1 |
| CA-miR5021 | ChiLCV_VAR_RVA | 702 | -22.7 |
| CA-miR5021 | ChiLCV_VAR_RVA | 696 | -17.4 |
| CA-miR5021 | ChiLCV_VAR_RVA | 696 | -20.6 |
| CA-miR5021 | ChiLCV_VAR_RVA | 702 | -16.4 |
| CA-miR2673a | ChiLCV_VAR_RVA | 2050 | -23.2 |
| CA-miR837-5p | ChiLCV_VAR_RVA | 2047 | -14.8 |
| CA-miR837-5p | ChiLCV_VAR_RVA | 2047 | -14.4 |
| CA-miR838 | ChiLCV_VAR_RVA | 2049 | -24.8 |
| CA-miR2673b | ChiLCV_VAR_RVA | 2050 | -23.2 |
| CA-miR5658 | ToLCNDV_RE_RVA/ | 1827 | -14 |
| CA-miR5021 | ToLCNDV_RE_RVA | 1545 | -25.6 |

**Table S4. List of predicted targeting sites by potential CA-miRNA within the begomovirus genome using the miRanda tool.**

| **miRNA** | **Begomovirus Isolate** | **Max Energy** | **Positions** |
| --- | --- | --- | --- |
| CA-miR837-5p | ChiLCV_KLD_01_RVA | -10.54 | 2047 |
| CA-miR837-5p | ChiLCV_Gkp | -1.87 | 2698 |
| CA-miR837-5p | ToLCNDV_RE_RVA | -3.07 | 675 |
| CA-miR837-5p | ToYLCV_DEO_RVA | -14.76 | 2071 |
| CA-miR837-5p | ChiLCV_GKP_RVA | -14.63 | 2047 |
| CA-miR837-5p | CLCuMuV_R_RVA | -18.97 | 2027 |
| CA-miR837-5p | ChiLCV_MZP_RVA | -14.63 | 2047 |
| CA-miR837-5p | ChiLCV_VAR_RVA | -14.63 | 2047 |
| CA-miR838 | ChiLCV_KLD_01_RVA | -15.97 | 2038 884 1603 |
| CA-miR838 | ChiLCV_Gkp | -15.89 | 2042 2056 |
| CA-miR838 | ChiLCV_KLD_RVA | -15.44 | 2039 884 |
| CA-miR838 | ToYLCV_DEO_RVA | -13.3 | 2056 |
| CA-miR838 | ChiLCV_GKP_RVA | -20.04 | 2043, 882 |
| CA-miR838 | ChiLCV_GZB_RVA | -15.89 | 2037 ,2051 |
| CA-miR838 | CLCuMuV_R_RVA | -19.83 | 2014 ,646 |
| CA-miR838 | ChiLCV_MZP_RVA | -20.04 | 2043, 882 |
| CA-miR838 | ChiLCV_VAR_RVA | -20.04 | 2043 ,882 |
| CA-miR5021 | ChiLCV_Gkp | -15.68 | 2525 ,706 |
| CA-miR5021 | ToLCNDV_RE_RVA | -19.27 | 1542 ,2167 |
| CA-miR5021 | ChiLCV_KLD_RVA | -15.26 | 2522 |
| CA-miR5021 | ToYLCV_DEO_RVA | -15.26 | 2533, 1558 |
| CA-miR5021 | ChiLCV_GZB_RVA | -12.32 | 2520 |
| CA-miR5658 | ChiLCV_Gkp | -12.4 | 933 |
| CA-miR5658 | ToLCNDV_RE_RVA | -24.21 | 817 |
| CA-miR5658 | ToYLCV_DEO_RVA | -14.57 | 944 |
| CA-miR5658 | ChiLCV_GZB_RVA | -16.38 | 314 |
| CA-miR5665 | ChiLCV_Gkp | -20.9 | 2661 |
| CA-miR5665 | ToLCNDV_RE_RVA | -17.81 | 124 |
| CA-miR1134 | ChiLCV_Gkp | -8.56 | 2284 |
| CA-miR1134 | ToLCNDV_RE_RVA | -12.86 | 1635, 700 |
| CA-miR1134 | ToYLCV_DEO_RVA | -6.22 | 1660 |
| CA-miR1134 | ChiLCV_GKP_RVA | -10.5 | 696 1 |
| CA-miR1134 | ChiLCV_GZB_RVA | -8.56 | 2279 |
| CA-miR1134 | ChiLCV_MZP_RVA | -10.5 | 696 |
| CA-miR1134 | ChiLCV_VAR_RVA | -5.57 | 696 |
| CA-miR1533 | ChiLCV_Gkp | -5.89 | 2487 |
| CA-miR1533 | ChiLCV_KLD_RVA | -6.65 | 1083 |
| CA-miR1533 | ToYLCV_DEO_RVA | -7.76 | 2495 |
| CA-miR1533 | ChiLCV_GKP_RVA | -15.1 | 1041 |
| CA-miR1533 | ChiLCV_MZP_RVA | -15.1 | 1041 |
| CA-miR1533 | ChiLCV_VAR_RVA | -15.1 | 10, 1041 |
| CA-miR1535 | ChiLCV_KLD_RVA | -12.82 | 677 |
| CA-miR1535 | CLCuMuV_R_RVA | -14.81 | 647 |
| CA-miR2108b | ChiLCV_KLD_01_RVA | -23.22 | 2597 |
| CA-miR2108b | ChiLCV_Gkp | -13.55 | 2059, 2127 |
| CA-miR2108b | ToLCNDV_RE_RVA | -14.06 | 2121 |
| CA-miR2108b | ChiLCV_KLD_RVA | -9.98 | 2124 |
| CA-miR2108b | ChiLCV_GKP_RVA | -8.77 | 2060 |
| CA-miR2108b | ChiLCV_GZB_RVA | -13.55 | 2054 |
| CA-miR2108b | ChiLCV_MZP_RVA | -8.77 | 2060 |
| CA-miR2108b | ChiLCV_VAR_RVA | -8.77 | 2060 |
| CA-miR3522 | ChiLCV_Gkp | -19.76 | 710 |
| CA-miR5041 | ChiLCV_KLD_01_RVA | -13.22 | 674, 49 |
| CA-miR5041 | ToLCNDV_RE_RVA | -16.84 | 1225, 92 |
| CA-miR5041 | ChiLCV_GKP_RVA | -14.42 | 672 |
| CA-miR5041 | ChiLCV_GZB_RVA | -14.42 | 674 |
| CA-miR5041 | CLCuMuV_R_RVA | -17.75 | 2337 |
| CA-miR5041 | ChiLCV_MZP_RVA | -14.42 | 672 |
| CA-miR5041 | ChiLCV_VAR_RVA | -14.42 | 672 |
| CA-miR2657a | ChiLCV_Gkp | -8.97 | 2559 |
| CA-miR2657a | ChiLCV_KLD_RVA | -5.89 | 2556 |
| CA-miR2657a | ChiLCV_GKP_RVA | -5.89 | 2558 |
| CA-miR2657a | ChiLCV_GZB_RVA | -5.89 | 2554 |
| CA-miR2657a | CLCuMuV_R_RVA | -7.12 | 49 |
| CA-miR2657a | ChiLCV_MZP_RVA | -5.89 | 2558 |
| CA-miR2657a | ChiLCV_VAR_RVA | -5.89 | 2558 |
| CA-miR2657b | ChiLCV_Gkp | -8.97 | 2559 |
| CA-miR2657b | ChiLCV_KLD_RVA | -5.89 | 2556 |
| CA-miR2657b | ChiLCV_GKP_RVA | -5.89 | 2558 |
| CA-miR2657b | ChiLCV_GZB_RVA | -5.89 | 2554 |
| CA-miR2657b | CLCuMuV_R_RVA | -7.12 | 49 |
| CA-miR2657b | ChiLCV_MZP_RVA | -5.89 | 2558 |
| CA-miR2657b | ChiLCV_VAR_RVA | -5.89 | 2558 |
| CA-miR2673a | ChiLCV_Gkp | -18.24 | 2054 |
| CA-miR2673a | ChiLCV_KLD_RVA | -22.08 | 2045 |
| CA-miR2673a | ToYLCV_DEO_RVA | -12.29 | 2057 |
| CA-miR2673a | ChiLCV_GKP_RVA | -18.89 | 2044 |
| CA-miR2673a | ChiLCV_GZB_RVA | -18.24 | 2049 |
| CA-miR2673a | CLCuMuV_R_RVA | -24.8 | 2024 |
| CA-miR2673a | ChiLCV_MZP_RVA | -18.89 | 2044 |
| CA-miR2673a | ChiLCV_VAR_RVA | -18.89 | 2044 |
| CA-miR2673b | ChiLCV_Gkp | -18.24 | 2054 |
| CA-miR2673b | ChiLCV_KLD_RVA | -22.08 | 2045 |
| CA-miR2673b | ToYLCV_DEO_RVA | -12.29 | 2057 |
| CA-miR2673b | ChiLCV_GKP_RVA | -18.89 | 2044 |
| CA-miR2673b | ChiLCV_GZB_RVA | -18.24 | 2049 |
| CA-miR2673b | CLCuMuV_R_RVA | -24.8 | 2024 |
| CA-miR2673b | ChiLCV_MZP_RVA | -18.89 | 2044 |
| CA-miR2673b | ChiLCV_VAR_RVA | -18.89 | 2044 |
| CA-miR395x | ChiLCV_KLD_01_RVA | -17.27 | 1188 |
| CA-miR414 | ToYLCV_DEO_RVA | -17.57 | 505, 664 ,1495 |
| CA-miR5565e | ChiLCV_KLD_01_RVA | -17.75 | 21 |
| CA-miR5565e | ChiLCV_Gkp | -14.58 | 1881 |
| CA-miR5565e | ToLCNDV_RE_RVA | -14.15 | 1876 |
| CA-miR5565e | ChiLCV_KLD_RVA | -11.84 | 1878 |
| CA-miR5565e | ToYLCV_DEO_RVA | -24.45 | 1898, 386 |
| CA-miR5565e | ChiLCV_GKP_RVA | -18.73 | 1880, 1507 |
| CA-miR5565e | ChiLCV_GZB_RVA | -14.58 | 1876 |
| CA-miR5565e | CLCuMuV_R_RVA | -14.58 | 1851 |
| CA-miR5565e | ChiLCV_MZP_RVA | -18.73 | 1880 ,1507 |
| CA-miR5565e | ChiLCV_VAR_RVA | -14.58 | 1880 |
| CA-miR2950 | ChiLCV_KLD_RVA | -14.94 | 38, 673 |
| CA-miR2950 | ChiLCV_GZB_RVA | -14.25 | 39 |
| CA-miR2950 | CLCuMuV_R_RVA | -17.56 | 644 |
| CA-miR2950 | ChiLCV_VAR_RVA | -14.25 | 39 |

**Table S5. List of predicted targeting sites by potential CA-miRNA within the begomovirus genome using the psRNATarget tool.**

| **miRNA_Acc.** | **Begomovirus Isolate** | **Expectation** | **Target_start** | **Target_end** | **Inhibition** |
| --- | --- | --- | --- | --- | --- |
| CA-miR837-5p | CLCuMuV_R_RVA | 2 | 2027 | 2047 | Cleavage |
| CA-miR838 | ChiLCV_MZP_RVA | 2 | 2052 | 2072 | Cleavage |
| CA-miR838 | ChiLCV_VAR_RVA | 2 | 2052 | 2072 | Cleavage |
| CA-miR838 | ChiLCV_GKP_RVA | 2 | 2052 | 2072 | Cleavage |
| CA-miR838 | CLCuMuV_R_RVA | 2 | 2023 | 2043 | Cleavage |
| CA-miR837-5p | ChiLCV_MZP_RVA | 3.5 | 2050 | 2070 | Cleavage |
| CA-miR837-5p | ChiLCV_VAR_RVA | 3.5 | 2050 | 2070 | Cleavage |
| CA-miR837-5p | ChiLCV_GKP_RVA | 3.5 | 2050 | 2070 | Cleavage |
| CA-miR837-5p | ToYLCV_DEO_RVA | 3.5 | 2071 | 2091 | Cleavage |
| CA-miR2108b | ChiLCV_KLD_01_RVA | 4 | 2597 | 2617 | Cleavage |
| CA-miR395x | ChiLCV_KLD_01_RVA | 4 | 1188 | 1208 | Cleavage |
| CA-miR837-5p | ChiLCV_GZB_RVA | 4 | 2052 | 2072 | Cleavage |
| CA-miR837-5p | ChiLCV_Gkp | 4 | 2057 | 2077 | Cleavage |
| CA-miR5021 | ToLCNDV_RE_RVA | 4.5 | 1542 | 1561 | Cleavage |
| CA-miR5041 | ToLCNDV_RE_RVA | 4.5 | 1602 | 1622 | Cleavage |
| CA-miR5658 | ToLCNDV_RE_RVA | 4.5 | 817 | 837 | Cleavage |
| CA-miR5041 | ToYLCV_DEO_RVA | 5 | 2224 | 2244 | Cleavage |
| CA-miR838 | ChiLCV_KLD_RVA | 5 | 2047 | 2068 | Cleavage |
| CA-miR1533 | ChiLCV_MZP_RVA | 5.5 | 1163 | 1181 | Cleavage |
| CA-miR1533 | ChiLCV_GZB_RVA | 5.5 | 1162 | 1180 | Cleavage |
| CA-miR1533 | ChiLCV_Gkp | 5.5 | 1167 | 1185 | Cleavage |
| CA-miR414 | CLCuMuV_R_RVA | 5.5 | 628 | 646 | Cleavage |
| CA-miR5041 | ChiLCV_KLD_RVA | 5.5 | 1607 | 1627 | Cleavage |
| CA-miR5041 | ChiLCV_Gkp | 5.5 | 1607 | 1627 | Cleavage |
| CA-miR5041 | ChiLCV_GZB_RVA | 5.5 | 1602 | 1622 | Cleavage |
| CA-miR5041 | CLCuMuV_R_RVA | 5.5 | 1577 | 1597 | Cleavage |
| CA-miR5041 | ChiLCV_GKP_RVA | 5.5 | 1606 | 1626 | Cleavage |
| CA-miR5041 | ChiLCV_MZP_RVA | 5.5 | 1606 | 1626 | Cleavage |
| CA-miR5565e | ChiLCV_KLD_RVA | 5.5 | 546 | 564 | Cleavage |
| CA-miR837-5p | ChiLCV_KLD_01_RVA | 5.5 | 2046 | 2066 | Cleavage |
| CA-miR2108b | ChiLCV_KLD_RVA | 6 | 2125 | 2144 | Cleavage |
| CA-miR2108b | ChiLCV_MZP_RVA | 6 | 1495 | 1514 | Cleavage |
| CA-miR2108b | ChiLCV_GKP_RVA | 6 | 1495 | 1514 | Cleavage |
| CA-miR2108b | ChiLCV_Gkp | 6 | 2128 | 2147 | Cleavage |
| CA-miR2657a | ChiLCV_KLD_RVA | 6 | 1493 | 1514 | Cleavage |
| CA-miR2657a | ChiLCV_KLD_RVA | 6 | 2372 | 2393 | Cleavage |
| CA-miR2657a | ChiLCV_MZP_RVA | 6 | 2374 | 2395 | Cleavage |
| CA-miR2657a | ChiLCV_VAR_RVA | 6 | 2374 | 2395 | Cleavage |
| CA-miR2657a | ChiLCV_GKP_RVA | 6 | 2374 | 2395 | Cleavage |
| CA-miR2657a | ChiLCV_GZB_RVA | 6 | 2370 | 2391 | Cleavage |
| CA-miR2657a | ChiLCV_Gkp | 6 | 2375 | 2396 | Cleavage |
| CA-miR2657a | CLCuMuV_R_RVA | 6 | 2529 | 2550 | Cleavage |
| CA-miR2657b | ChiLCV_KLD_RVA | 6 | 1493 | 1514 | Cleavage |
| CA-miR2657b | ChiLCV_KLD_RVA | 6 | 2372 | 2393 | Cleavage |
| CA-miR2657b | ChiLCV_MZP_RVA | 6 | 2374 | 2395 | Cleavage |
| CA-miR2657b | ChiLCV_VAR_RVA | 6 | 2374 | 2395 | Cleavage |
| CA-miR2657b | ChiLCV_GKP_RVA | 6 | 2374 | 2395 | Cleavage |
| CA-miR2657b | ChiLCV_GZB_RVA | 6 | 2370 | 2391 | Cleavage |
| CA-miR2657b | ChiLCV_Gkp | 6 | 2375 | 2396 | Cleavage |
| CA-miR2657b | CLCuMuV_R_RVA | 6 | 2529 | 2550 | Cleavage |
| CA-miR395x | ToYLCV_DEO_RVA | 6 | 1212 | 1232 | Cleavage |
| CA-miR414 | ChiLCV_GKP_RVA | 6 | 657 | 675 | Cleavage |
| CA-miR414 | ChiLCV_VAR_RVA | 6 | 657 | 675 | Cleavage |
| CA-miR414 | ChiLCV_MZP_RVA | 6 | 657 | 675 | Cleavage |
| CA-miR5041 | ToYLCV_DEO_RVA | 6 | 1624 | 1644 | Cleavage |
| CA-miR5041 | ChiLCV_VAR_RVA | 6 | 1606 | 1626 | Cleavage |
| CA-miR5658 | ChiLCV_MZP_RVA | 6 | 2279 | 2299 | Cleavage |
| CA-miR5658 | ChiLCV_GKP_RVA | 6 | 2279 | 2299 | Cleavage |
| CA-miR5658 | ChiLCV_KLD_01_RVA | 6 | 2278 | 2298 | Cleavage |
| CA-miR837-5p | ChiLCV_KLD_RVA | 6 | 2047 | 2067 | Cleavage |
| CA-miR837-5p | ChiLCV_KLD_01_RVA | 6 | 2558 | 2578 | Cleavage |
| CA-miR1533 | ChiLCV_VAR_RVA | 6.5 | 1166 | 1184 | Cleavage |
| CA-miR1533 | ChiLCV_GKP_RVA | 6.5 | 1166 | 1184 | Cleavage |
| CA-miR2108b | ChiLCV_KLD_RVA | 6.5 | 1496 | 1515 | Cleavage |
| CA-miR2108b | ChiLCV_VAR_RVA | 6.5 | 72 | 92 | Cleavage |
| CA-miR2108b | ChiLCV_VAR_RVA | 6.5 | 462 | 482 | Cleavage |
| CA-miR2108b | ToLCNDV_RE_RVA | 6.5 | 2122 | 2142 | Cleavage |
| CA-miR2657a | ChiLCV_GZB_RVA | 6.5 | 1488 | 1509 | Cleavage |
| CA-miR2657b | ChiLCV_GZB_RVA | 6.5 | 1488 | 1509 | Cleavage |
| CA-miR5041 | ChiLCV_KLD_01_RVA | 6.5 | 1605 | 1625 | Cleavage |
| CA-miR5565e | ChiLCV_MZP_RVA | 6.5 | 1975 | 1993 | Cleavage |
| CA-miR5565e | ChiLCV_VAR_RVA | 6.5 | 1975 | 1993 | Cleavage |
| CA-miR5565e | ChiLCV_GKP_RVA | 6.5 | 1975 | 1993 | Cleavage |
| CA-miR5658 | ChiLCV_KLD_01_RVA | 6.5 | 817 | 837 | Cleavage |
| CA-miR5658 | ChiLCV_KLD_01_RVA | 6.5 | 2156 | 2176 | Cleavage |
| CA-miR5658 | ChiLCV_GZB_RVA | 6.5 | 721 | 741 | Cleavage |
| CA-miR5658 | CLCuMuV_R_RVA | 6.5 | 786 | 806 | Cleavage |
| CA-miR837-5p | ToLCNDV_RE_RVA | 6.5 | 2044 | 2064 | Cleavage |
| CA-miR1134 | ToLCNDV_RE_RVA | 7 | 700 | 723 | Cleavage |
| CA-miR1134 | CLCuMuV_R_RVA | 7 | 241 | 264 | Cleavage |
| CA-miR1533 | ToLCNDV_RE_RVA | 7 | 1921 | 1939 | Cleavage |
| CA-miR1533 | ChiLCV_KLD_01_RVA | 7 | 1162 | 1180 | Cleavage |
| CA-miR1535 | ChiLCV_KLD_01_RVA | 7 | 1696 | 1714 | Cleavage |
| CA-miR1535 | ChiLCV_VAR_RVA | 7 | 113 | 131 | Cleavage |
| CA-miR2108b | ChiLCV_KLD_01_RVA | 7 | 2123 | 2143 | Cleavage |
| CA-miR2108b | ToYLCV_DEO_RVA | 7 | 2142 | 2161 | Cleavage |
| CA-miR2108b | ToYLCV_DEO_RVA | 7 | 480 | 500 | Cleavage |
| CA-miR2108b | ChiLCV_KLD_RVA | 7 | 463 | 483 | Cleavage |
| CA-miR2108b | ChiLCV_MZP_RVA | 7 | 462 | 482 | Cleavage |
| CA-miR2108b | ChiLCV_GKP_RVA | 7 | 462 | 482 | Cleavage |
| CA-miR2108b | ChiLCV_GZB_RVA | 7 | 1491 | 1510 | Cleavage |
| CA-miR2108b | ChiLCV_Gkp | 7 | 466 | 486 | Cleavage |
| CA-miR2108b | ToLCNDV_RE_RVA | 7 | 1986 | 2007 | Cleavage |
| CA-miR2108b | ToLCNDV_RE_RVA | 7 | 464 | 484 | Cleavage |
| CA-miR2108b | CLCuMuV_R_RVA | 7 | 889 | 909 | Cleavage |
| CA-miR2108b | CLCuMuV_R_RVA | 7 | 232 | 252 | Cleavage |
| CA-miR2108b | CLCuMuV_R_RVA | 7 | 433 | 453 | Cleavage |
| CA-miR2657a | ToYLCV_DEO_RVA | 7 | 2222 | 2244 | Cleavage |
| CA-miR2657a | ToYLCV_DEO_RVA | 7 | 2383 | 2404 | Cleavage |
| CA-miR2657b | ToYLCV_DEO_RVA | 7 | 2222 | 2244 | Cleavage |
| CA-miR2657b | ToYLCV_DEO_RVA | 7 | 2383 | 2404 | Cleavage |
| CA-miR2950 | ToYLCV_DEO_RVA | 7 | 2382 | 2402 | Cleavage |
| CA-miR395x | ChiLCV_GZB_RVA | 7 | 82 | 102 | Cleavage |
| CA-miR395x | ChiLCV_Gkp | 7 | 82 | 102 | Cleavage |
| CA-miR414 | ChiLCV_KLD_01_RVA | 7 | 659 | 677 | Cleavage |
| CA-miR5041 | ChiLCV_Gkp | 7 | 258 | 277 | Cleavage |
| CA-miR5565e | CLCuMuV_R_RVA | 7 | 1313 | 1331 | Cleavage |
| CA-miR5565e | CLCuMuV_R_RVA | 7 | 1969 | 1987 | Cleavage |
| CA-miR5565e | ToLCNDV_RE_RVA | 7 | 2666 | 2684 | Cleavage |
| CA-miR5658 | ChiLCV_GZB_RVA | 7 | 817 | 837 | Cleavage |
| CA-miR5658 | ToYLCV_DEO_RVA | 7 | 833 | 853 | Cleavage |
| CA-miR837-5p | ChiLCV_KLD_RVA | 7 | 1491 | 1511 | Cleavage |
| CA-miR5015b | ChiLCV_Gkp | 4.5 | 1175 | 1195 | Cleavage |
| CA-miR5015b | ChiLCV_VAR_RVA | 5 | 1174 | 1194 | Cleavage |
| CA-miR5015b | ChiLCV_GKP_RVA | 5 | 1174 | 1194 | Cleavage |
| CA-miR5015b | ChiLCV_GZB_RVA | 5.5 | 1170 | 1190 | Cleavage |
| CA-miR5015b | ChiLCV_MZP_RVA | 5.5 | 1171 | 1191 | Cleavage |
| CA-miR5015b | ChiLCV_Gkp | 6 | 1505 | 1525 | Cleavage |
| CA-miR5015b | ChiLCV_GKP_RVA | 6 | 39 | 59 | Cleavage |
| CA-miR5015b | ChiLCV_MZP_RVA | 6 | 39 | 59 | Cleavage |
| CA-miR5015b | ChiLCV_VAR_RVA | 6.5 | 1504 | 1524 | Cleavage |
| CA-miR5015b | ChiLCV_GZB_RVA | 6.5 | 1500 | 1520 | Cleavage |
| CA-miR5015b | ToLCNDV_RE_RVA | 6.5 | 1500 | 1520 | Cleavage |
| CA-miR5015b | ToLCNDV_RE_RVA | 6.5 | 795 | 815 | Cleavage |
| CA-miR5015b | ChiLCV_KLD_RVA | 6.5 | 1505 | 1525 | Cleavage |
| CA-miR5015b | ToLCNDV_RE_RVA | 7 | 40 | 60 | Cleavage |
| CA-miR5015b | CLCuMuV_R_RVA | 7 | 1036 | 1056 | Cleavage |

**Table S6. List of predicted targeting sites by potential CA-miRNA within the begomovirus genome using the RNA22 tool.**

| **miR Name** | **transcript name** | **leftmost position of** | **folding energy** | **heteroduplex** | **p value** |
| --- | --- | --- | --- | --- | --- |
|  |  | **predicted target site** | **(in -Kcal/mol)** |  |  |
| CA_miR5665 | ToYLCV_DEO_RVA | 807 | -15.4 | AGCCTTCTACT--GCCACTAT | 1.66E-01 |
|  |  |  |  | \|\|:\|\| \|\| \|\|\|\|:\|: |  |
|  |  |  |  | AAGGGAGTAGAACAGGTGGTG |  |
|  |  |  |  |  |  |
| CA_miR5665 | ToYLCV_DEO_RVA | 1429 | -12.7 | CTCCCAGAT-AAAAACGCCAT | 3.28E-01 |
|  |  |  |  | \|\|\|\| \|\| \|:\|\|\|: |  |
|  |  |  |  | AAGGGAGTAGAACAGGTGGTG |  |
|  |  |  |  |  |  |
| CA_miR5665 | ToYLCV_DEO_RVA | 1794 | -16.9 | GTCCCTC--TGGG-CCTCCAT | 6.74E-03 |
|  |  |  |  | \|\|\|\|\|\| \| \|\| \|\|\|: |  |
|  |  |  |  | AAGGGAGTAGAACAGGTGGTG |  |
|  |  |  |  |  |  |
| CA_miR5665 | ToYLCV_DEO_RVA | 1954 | -13.7 | TTGCC-GGTCCTG-CTATCAC | 7.87E-03 |
|  |  |  |  | \|\| \|\| :\|\| \|\| \|:\|:\|\|\| |  |
|  |  |  |  | AAGGGAGTAGAACAGGTGGTG |  |
|  |  |  |  |  |  |
| CA_miR5665 | ToYLCV_DEO_RVA | 1960 | -12.8 | GTCCTGCTATCACCCTCTATTAC | 7.87E-03 |
|  |  |  |  | \|\|\|: :\|\|\| \|\|:\|::\|\| |  |
|  |  |  |  | AAGGG-AGTAG-AACAGGTGGTG |  |
|  |  |  |  |  |  |
| CA_miR837_5p | ToYLCV_DEO_RVA | 2062 | -12.4 | TTAAAAGAAGAAGATAAAAAG | 2.17E-01 |
|  |  |  |  | \|\|\|\|:\|\|:\|\|\|\| \|\|\|\|\|\| |  |
|  |  |  |  | ATTTTTTTTTTTCTTTTTTTC |  |
|  |  |  |  |  |  |
| CA_miR837_5p | ToYLCV_DEO_RVA | 2071 | -12.6 | GAAGATAAAAAGGGAGAAATG | 2.17E-01 |
|  |  |  |  | \|\|:\|:\|:\|\|\|\|: |  |
|  |  |  |  | ATTTTTTTTTTTCTTTTTTAT |  |
|  |  |  |  |  |  |
| CA_miR5041 | ToYLCV_DEO_RVA | 2635 | -14.3 | ATGAGTACCGAT--TGACCAAG |  |
|  |  |  |  | \|\|\|\|:\| \|\|\| \|\|\|\|\|\|: |  |
|  |  |  |  | CACTCGT-TCTACTTCTGGTTT | 9.15E-02 |
|  |  |  |  |  |  |
| CA_miR5658 | ChiLCV_Gkp | 361 | -12 | GC-CGCCGTC-TCAACTTCGA | 1.71E-01 |
|  |  |  |  | \|\| \|: \|:\|\| \|\|\| \|:\|\|: |  |
|  |  |  |  | CGAGTAGTAGTAGTAGGAGTG |  |
|  |  |  |  |  |  |
| CA_miR3522 | ChiLCV_Gkp | 711 | -17.3 | GTC-ATGTTCTTTTTGGTCC | 2.52E-01 |
|  |  |  |  | \|\| \|\|:\|\| \|\|\|\|\|\|\|\| |  |
|  |  |  |  | AAGGGACGAG-TAAACCAGG |  |
|  |  |  |  |  |  |
| CA_miR5658 | ChiLCV_Gkp | 1137 | -13 | ACATTCCATAATACATGATCAA | 1.61E-01 |
|  |  |  |  | \|\|\|\|\|\|\| \|\|\| \|\|\|\| |  |
|  |  |  |  | CTAGTGGTATTA-GTAGTAGTA |  |
|  |  |  |  |  |  |
| CA_miR5658 | ChiLCV_Gkp | 1226 | -13 | AAATACCCTTAAGAAATCACCAG | 4.19E-03 |
|  |  |  |  | \|\|\|:\| \|\|\|\|\|\|\| |  |
|  |  |  |  | AAAGTAGGAGT--GATAGTGGTA |  |
|  |  |  |  |  |  |
| CA_miR5665 | ChiLCV_Gkp | 1229 | -13.1 | TACCCTTA-AGAAATCACCAG | 4.19E-03 |
|  |  |  |  | \|\|\|\|:\| :\|\|\|\|\| |  |
|  |  |  |  | AAGGGAGTAGAACAGGTGGTG |  |
|  |  |  |  |  |  |
| CA_miR5021 | ChiLCV_Gkp | 1545 | -12.5 | CCT-GGTCCCCTTCTTGGCC | 2.56E-01 |
|  |  |  |  | \|\| \|\| \|\|\|\|\|\| :\|\| |  |
|  |  |  |  | AGAAGAAGAAGAAGAAGTGG |  |
|  |  |  |  |  |  |
| CA_miR5665 | ChiLCV_Gkp | 1777 | -14.3 | GTCCCTT--TGGG-CCCCCAT | 1.49E-01 |
|  |  |  |  | \|\|\|\|\|: \| \|\| \|\|\|: |  |
|  |  |  |  | AAGGGAGTAGAACAGGTGGTG |  |
|  |  |  |  |  |  |
| CA_miR5658 | ChiLCV_Gkp | 2010 | -12.3 | TCTCGGCAGCCCATTCCTCAA | 4.42E-02 |
|  |  |  |  | \|\|\|: \|\| \|\|\|\|\|\| |  |
|  |  |  |  | CGAGTAGTAGTAGTAGGAGTG |  |
|  |  |  |  |  |  |
| CA_miR5658 | ChiLCV_Gkp | 2018 | -12.7 | GCCCATTCCTCAAGTTCCTCGG | 4.42E-02 |
|  |  |  |  | \|\| \|\|\|: \|\|\| \|\|\|\|\|: |  |
|  |  |  |  | CGAGTAG-TAGTAGTAGGAGTG |  |
|  |  |  |  |  |  |
| CA_miR838 | ChiLCV_Gkp | 2053 | -14.4 | AGAAGAAGAACAAAAAGGAGA | 2.32E-02 |
|  |  |  |  | \|\|\|\|\|\|\|\| \|\| \|\|\|:\|\|\| |  |
|  |  |  |  | ACTTCTTCTTCTTCTTCTTCT |  |
|  |  |  |  |  |  |
| CA_miR5658 | ChiLCV_GKP_RVA | 357 | -12 | GC-CGCCGTC-TCAACTTCGA | 1.63E-01 |
|  |  |  |  | \|\| \|: \|:\|\| \|\|\| \|:\|\|: |  |
|  |  |  |  | CGAGTAGTAGTAGTAGGAGTG |  |
|  |  |  |  |  |  |
| CA_miR5658 | ChiLCV_GKP_RVA | 406 | -13.4 | CATTGTCC--GC-GTCACCAA | 1.91E-01 |
|  |  |  |  | \| \|\|\| :\| :\|\|\|\|\|\| |  |
|  |  |  |  | AAAGTAGGAGTGATAGTGGTA |  |
|  |  |  |  |  |  |
| CA_miR2657a | ChiLCV_GKP_RVA | 833 | -12.2 | CAGG-TGC-TGAGGAAGTGGCA | 2.60E-01 |
|  |  |  |  | \|\|:\| :\| \|\| \|\|\|:\|::\|\| |  |
|  |  |  |  | GTTCTTTGTTCTACTTTATTGT |  |
|  |  |  |  |  |  |
| CA_miR2657b | ChiLCV_GKP_RVA | 833 | -12.2 | CAGG-TGC-TGAGGAAGTGGCA | 2.60E-01 |
|  |  |  |  | \|\|:\| :\| \|\| \|\|\|:\|::\|\| |  |
|  |  |  |  | GTTCTTTGTTCTACTTTATTGT |  |
|  |  |  |  |  |  |
| CA_miR5665 | ChiLCV_GKP_RVA | 1417 | -12.7 | CTCCCAGAT-AAACACGCCAT | 2.80E-01 |
|  |  |  |  | \|\|\|\| \|\| \|:\|\|\|: |  |
|  |  |  |  | AAGGGAGTAGAACAGGTGGTG |  |
|  |  |  |  |  |  |
| CA_miR5021 | ChiLCV_GKP_RVA | 1543 | -12.3 | GCCTGATC--CC-CTTCTTG | 1.88E-01 |
|  |  |  |  | \|\|\|\|\| \|\| \|\|\|\|\|\|: |  |
|  |  |  |  | AAAACTAGAAGGAGAAGAAT |  |
|  |  |  |  |  |  |
| CA_miR5665 | ChiLCV_GKP_RVA | 1635 | -13.7 | AGCCCAATTCTTGAGTGCGCTAT | 3.88E-01 |
|  |  |  |  | \|\|\| \|\|\|\| \|\| \|:\|:\|: |  |
|  |  |  |  | AAGGGAGTAGAA--CAGGTGGTG |  |
|  |  |  |  |  |  |
| CA_miR837_5p | ChiLCV_GKP_RVA | 2050 | -13.4 | AAAGAAGAAGAAGAAAAAGGA | 1.34E-03 |
|  |  |  |  | \|\|:\|\|:\|\|:\|\|\|\|\|\|\|\|:: |  |
|  |  |  |  | ATTTTTTTTTTTCTTTTTTTC |  |
|  |  |  |  |  |  |
| CA_miR838 | ChiLCV_GKP_RVA | 2052 | -23.5 | AGAAGAAGAAGAAAAAGGAGA | 1.34E-03 |
|  |  |  |  | \|\|\|\|\|\|\|\|\|\|\|\| \|\|\|:\|\|\| |  |
|  |  |  |  | ACTTCTTCTTCTTCTTCTTCT |  |
|  |  |  |  |  |  |
| CA_miR2108b | ChiLCV_GKP_RVA | 2060 | -12.4 | AAGAAAAAGGAGAAACATAAA | 1.34E-03 |
|  |  |  |  | \|\|\| : :\|\|\|\|\|\|\|\|\| |  |
|  |  |  |  | TATTGTTTGTGTTTTGTATTT |  |
|  |  |  |  |  |  |
| CA_miR5658 | ChiLCV_GKP_RVA | 2286 | -12.7 | GT-AATCACCGTCCTTCTCGA | 1.07E-01 |
|  |  |  |  | \|: \|\|\|\| \|:\|\| \|:\|\|\|: |  |
|  |  |  |  | CGAGTAGTAGTAGTAGGAGTG |  |
|  |  |  |  |  |  |
| CA_miR5041 | ChiLCV_KLD_01_RVA | 655 | -19.2 | TTGGGCAAGATCTGGATGGAA | 3.84E-01 |
|  |  |  |  | \|\|:\|\|\|\|\|\|\| :\|\|: :\|\| |  |
|  |  |  |  | CACTCGTTCTACTTCTGGTTT |  |
|  |  |  |  |  |  |
| CA_miR2673a | ChiLCV_KLD_01_RVA | 656 | -13.3 | TGGGCAAGATCTGGATGGAAGAGA | 3.84E-01 |
|  |  |  |  | ::\| \|\|\|\| :\|\| :\|\|\|\|\|\| |  |
|  |  |  |  | CTTC-TTCTTCTTCT-TCTTCTCC |  |
|  |  |  |  |  |  |
| CA_miR2673b | ChiLCV_KLD_01_RVA | 656 | -13.3 | TGGGCAAGATCTGGATGGAAGAGA | 3.84E-01 |
|  |  |  |  | ::\| \|\|\|\| :\|\| :\|\|\|\|\|\| |  |
|  |  |  |  | CTTC-TTCTTCTTCT-TCTTCTCC |  |
|  |  |  |  |  |  |
| CA_miR5658 | ChiLCV_KLD_01_RVA | 850 | -15.9 | CGGCATGC-AACTGTCACCGG | 3.74E-01 |
|  |  |  |  | \|\|\| \| \|\|\|:\|\|\|\|\|: |  |
|  |  |  |  | AAAGTAGGAGTGATAGTGGTA |  |
|  |  |  |  |  |  |
| CA_miR5658 | ChiLCV_KLD_01_RVA | 1992 | -12.3 | GCGGAACCGATCA-CATTTTCAC | 2.17E-01 |
|  |  |  |  | \|\| \|\|\|\| \|\|\|::\|\|\|\| |  |
|  |  |  |  | CG--AGTAGTAGTAGTAGGAGTG |  |
|  |  |  |  |  |  |
| CA_miR5658 | ChiLCV_KLD_RVA | 358 | -12 | GC-CGCCGTC-TCAACTTCGA | 1.71E-01 |
|  |  |  |  | \|\| \|: \|:\|\| \|\|\| \|:\|\|: |  |
|  |  |  |  | CGAGTAGTAGTAGTAGGAGTG |  |
|  |  |  |  |  |  |
| CA_miR5665 | ChiLCV_KLD_RVA | 1418 | -12.7 | CTCCCAGAT-AAACACGCCAT | 2.64E-01 |
|  |  |  |  | \|\|\|\| \|\| \|:\|\|\|: |  |
|  |  |  |  | AAGGGAGTAGAACAGGTGGTG |  |
|  |  |  |  |  |  |
| CA_miR2673a | ChiLCV_KLD_RVA | 2048 | -19.6 | AAAGAAGAAGATAGAAAAGGAGA | 1.02E-01 |
|  |  |  |  | \|\|\|\|\|\|\|\|\|\| \|\|\|\| \|:\|\|\| |  |
|  |  |  |  | CTTCTTCTTCT-TCTTCTTCTCC |  |
|  |  |  |  |  |  |
| CA_miR2673b | ChiLCV_KLD_RVA | 2048 | -19.6 | AAAGAAGAAGATAGAAAAGGAGA | 1.02E-01 |
|  |  |  |  | \|\|\|\|\|\|\|\|\|\| \|\|\|\| \|:\|\|\| |  |
|  |  |  |  | CTTCTTCTTCT-TCTTCTTCTCC |  |
|  |  |  |  |  |  |
| CA_miR837_5p | ChiLCV_KLD_RVA | 2049 | -13.1 | AAGAAGAAGATAGAAAAGGAG | 1.02E-01 |
|  |  |  |  | \|:\|\|:\|\|:\| \|\|\|\|\|\|::\|\| |  |
|  |  |  |  | ATTTTTTTTTTTCTTTTTTTC |  |
|  |  |  |  |  |  |
| CA_miR838 | ChiLCV_KLD_RVA | 2050 | -18.5 | AGAAGAAGATAGAA-AAGGAGA | 1.02E-01 |
|  |  |  |  | \|\|\|\|\|\|\|\| \|\|\|\| \|\|\|:\|\|\| |  |
|  |  |  |  | ACTTCTTCT-TCTTCTTCTTCT |  |
|  |  |  |  |  |  |
| CA_miR5665 | CLCuMuV_R_RVA | 109 | -12.6 | TTCAAACATGTGGGATCCACTAT | 3.26E-02 |
|  |  |  |  | \|\|\| \|\|\| \| \| \|\|\|\|\|:\|: |  |
|  |  |  |  | AAGGGAGTAGA-AC-AGGTGGTG |  |
|  |  |  |  |  |  |
| CA_miR5658 | CLCuMuV_R_RVA | 328 | -12 | GC-CGCCGTC-TCAACTTCGA | 1.66E-01 |
|  |  |  |  | \|\| \|: \|:\|\| \|\|\| \|:\|\|: |  |
|  |  |  |  | CGAGTAGTAGTAGTAGGAGTG |  |
|  |  |  |  |  |  |
| CA_miR5658 | CLCuMuV_R_RVA | 377 | -13.4 | CATTGTCC--GC-GTCACCAA | 1.88E-01 |
|  |  |  |  | \| \|\|\| :\| :\|\|\|\|\|\| |  |
|  |  |  |  | AAAGTAGGAGTGATAGTGGTA |  |
|  |  |  |  |  |  |
| CA_miR5658 | CLCuMuV_R_RVA | 819 | -14.3 | TGGTATGC-AACTGTCACCGG | 1.10E-01 |
|  |  |  |  | :\|\| \| \|\|\|:\|\|\|\|\|: |  |
|  |  |  |  | AAAGTAGGAGTGATAGTGGTA |  |
|  |  |  |  |  |  |
| CA_miR5021 | CLCuMuV_R_RVA | 1505 | -15.1 | CTCTCCTCCTC--CTGTTGC | 4.18E-02 |
|  |  |  |  | \|\|\|\| \|\|\|\|\| \|\| :\|\|\| |  |
|  |  |  |  | AAGAGAAGGAGAAGAAGACG |  |
|  |  |  |  |  |  |
| CA_miR5658 | CLCuMuV_R_RVA | 1920 | -13.3 | TATC-TCCTT-CAATCACTAT | 7.27E-02 |
|  |  |  |  | \|\| \|\|\|\|: \| \|\|\|\|\|:\|\| |  |
|  |  |  |  | AAAGTAGGAGTGATAGTGGTA |  |
|  |  |  |  |  |  |
| CA_miR5658 | CLCuMuV_R_RVA | 1964 | -14.3 | GCGGCATCGACAACGTTTTCGG | 9.00E-02 |
|  |  |  |  | \|\| \|\|\|\|: \|\| \|:\|::\|\|: |  |
|  |  |  |  | CG-AGTAGTAGTAGTAGGAGTG |  |
|  |  |  |  |  |  |
| CA_miR5021 | CLCuMuV_R_RVA | 1991 | -17.1 | CACTCTTCAAGTTCTTCTGG | 9.00E-02 |
|  |  |  |  | \|\|\|\|\|\| \|\|\|\|\|\|\|\| |  |
|  |  |  |  | AAGAGAAGGAGAAGAAGACG |  |
|  |  |  |  |  |  |
| CA_miR837_5p | CLCuMuV_R_RVA | 2018 | -13.6 | TCGAAAGAAGAAGAAGAAAAA | 1.82E-03 |
|  |  |  |  | :\|\|\|:\|\|:\|\|\|\|\|:\|\|\|\| |  |
|  |  |  |  | ATTTTTTTTTTTCTTTTTTTC |  |
|  |  |  |  |  |  |
| CA_miR838 | CLCuMuV_R_RVA | 2020 | -20.2 | GAAAGAAGAAGAAGAAAAAGG | 1.82E-03 |
|  |  |  |  | \|\|\|\|\|\|\|\|\|\|\|\|\|\| \|\|\|: |  |
|  |  |  |  | ACTTCTTCTTCTTCTTCTTCT |  |
|  |  |  |  |  |  |
| CA_miR5665 | ToLCNDV_RE_RVA | 125 | -14 | CACC-AAGTTTGGATCCACCAA | 2.72E-01 |
|  |  |  |  | \|\| :\|:\| \|\|\|\|\|\|\| |  |
|  |  |  |  | AAGGGAGTAGA-ACAGGTGGTG |  |
|  |  |  |  |  |  |
| CA_miR5658 | ToLCNDV_RE_RVA | 359 | -12 | GC-CGACGTC-TCAACTTCGA | 3.55E-01 |
|  |  |  |  | \|\| \|: \|:\|\| \|\|\| \|:\|\|: |  |
|  |  |  |  | CGAGTAGTAGTAGTAGGAGTG |  |
|  |  |  |  |  |  |
| CA_miR3522 | ToLCNDV_RE_RVA | 709 | -12.6 | GTC-ATGTTTTTTTTGGTTC | 8.87E-02 |
|  |  |  |  | \|\| \|\|:\|: \|\|\|\|\|\|:\| |  |
|  |  |  |  | AAGGGACGAG-TAAACCAGG |  |
|  |  |  |  |  |  |
| CA_miR5658 | ToLCNDV_RE_RVA | 813 | -12 | GAACATGCATCGTGATCGTTAT | 1.61E-01 |
|  |  |  |  | \|\|\| \|\| \|:\|:\|\|\|:\|:\| |  |
|  |  |  |  | AAAGTA-GTTGTATTAGTAGTT |  |
|  |  |  |  |  |  |
| CA_miR5658 | ToLCNDV_RE_RVA | 813 | -12.8 | GAACATGCATCGTGATCGTTAT | 1.61E-01 |
|  |  |  |  | \|\|\| \|\|\|\|:\| \|\|\| \|:\| |  |
|  |  |  |  | CGAGTA-GTAGTAGTAGGAGTG |  |
|  |  |  |  |  |  |
| CA_miR5041 | ToLCNDV_RE_RVA | 1225 | -12.9 | ACTCTCAAGA-AAAGACCAGT | 2.52E-01 |
|  |  |  |  | \|\|\|\|\| \|\|\|\|\|\|\|: |  |
|  |  |  |  | CACTCGTTCTACTTCTGGTTT |  |
|  |  |  |  |  |  |
| CA_miR5021 | ToLCNDV_RE_RVA | 1542 | -17.2 | GAATGCTCTTCTTCTTCTTC | 3.89E-03 |
|  |  |  |  | \|\| \|\|\|\|\|:\|\|\|\|\|\|\| |  |
|  |  |  |  | AAAACTAGAAGGAGAAGAAT |  |
|  |  |  |  |  |  |
| CA_miR5021 | ToLCNDV_RE_RVA | 1543 | -17.9 | AATGCT-CTTCTTCTTCTTC | 3.89E-03 |
|  |  |  |  | \|\| \|\|\|\|\|\|\|:\|\|\|\|\| |  |
|  |  |  |  | AAAAGATGAAGAAGGAGAAG |  |
|  |  |  |  |  |  |
| CA_miR5021 | ToLCNDV_RE_RVA | 1545 | -25.9 | TGCTCTTCTTCTTCTTCTGG | 3.89E-03 |
|  |  |  |  | \|\|\|\|\|\|:\|\|\|\|\|\|\|\|\|\| |  |
|  |  |  |  | AAGAGAAGGAGAAGAAGACG |  |
|  |  |  |  |  |  |
| CA_miR5021 | ToLCNDV_RE_RVA | 1644 | -13.4 | AGTGCTGCATTTTTTTCTTC | 7.17E-02 |
|  |  |  |  | \|\|:\| \|:\|\|::\|\|\|\|\| |  |
|  |  |  |  | AAAAGATGAAGAAGGAGAAG |  |
|  |  |  |  |  |  |
| CA_miR5665 | ToLCNDV_RE_RVA | 2222 | -16.6 | GGCCGTCTGCT-GACCACCAC | 2.72E-01 |
|  |  |  |  | \|\| \|\| \|\| \|\|\|\|\|\|\| |  |
|  |  |  |  | AAGGGAGTAGAACAGGTGGTG |  |
|  |  |  |  |  |  |
| CA_miR5665 | ToLCNDV_RE_RVA | 125 | -14 | CACC-AAGTTTGGATCCACCAA | 2.72E-01 |
|  |  |  |  | \|\| :\|:\| \|\|\|\|\|\|\| |  |
|  |  |  |  | AAGGGAGTAGA-ACAGGTGGTG |  |
|  |  |  |  |  |  |
| CA_miR5658 | ToLCNDV_RE_RVA | 359 | -12 | GC-CGACGTC-TCAACTTCGA | 3.55E-01 |
|  |  |  |  | \|\| \|: \|:\|\| \|\|\| \|:\|\|: |  |
|  |  |  |  | CGAGTAGTAGTAGTAGGAGTG |  |
|  |  |  |  |  |  |
| CA_miR3522 | ToLCNDV_RE_RVA | 709 | -12.6 | GTC-ATGTTTTTTTTGGTTC | 8.87E-02 |
|  |  |  |  | \|\| \|\|:\|: \|\|\|\|\|\|:\| |  |
|  |  |  |  | AAGGGACGAG-TAAACCAGG |  |
|  |  |  |  |  |  |
| CA_miR5658 | ToLCNDV_RE_RVA | 813 | -12 | GAACATGCATCGTGATCGTTAT | 1.61E-01 |
|  |  |  |  | \|\|\| \|\| \|:\|:\|\|\|:\|:\| |  |
|  |  |  |  | AAAGTA-GTTGTATTAGTAGTT |  |
|  |  |  |  |  |  |
| CA_miR5658 | ToLCNDV_RE_RVA | 813 | -12.8 | GAACATGCATCGTGATCGTTAT | 1.61E-01 |
|  |  |  |  | \|\|\| \|\|\|\|:\| \|\|\| \|:\| |  |
|  |  |  |  | CGAGTA-GTAGTAGTAGGAGTG |  |
|  |  |  |  |  |  |
| CA_miR5041 | ToLCNDV_RE_RVA | 1225 | -12.9 | ACTCTCAAGA-AAAGACCAGT | 2.52E-01 |
|  |  |  |  | \|\|\|\|\| \|\|\|\|\|\|\|: |  |
|  |  |  |  | CACTCGTTCTACTTCTGGTTT |  |
|  |  |  |  |  |  |
| CA_miR5021 | ToLCNDV_RE_RVA | 1542 | -17.2 | GAATGCTCTTCTTCTTCTTC | 3.89E-03 |
|  |  |  |  | \|\| \|\|\|\|\|:\|\|\|\|\|\|\| |  |
|  |  |  |  | AAAACTAGAAGGAGAAGAAT |  |
|  |  |  |  |  |  |
| CA_miR5021 | ToLCNDV_RE_RVA | 1543 | -17.9 | AATGCT-CTTCTTCTTCTTC | 3.89E-03 |
|  |  |  |  | \|\| \|\|\|\|\|\|\|:\|\|\|\|\| |  |
|  |  |  |  | AAAAGATGAAGAAGGAGAAG |  |
|  |  |  |  |  |  |
| CA_miR5021 | ToLCNDV_RE_RVA | 1545 | -25.9 | TGCTCTTCTTCTTCTTCTGG | 3.89E-03 |
|  |  |  |  | \|\|\|\|\|\|:\|\|\|\|\|\|\|\|\|\| |  |
|  |  |  |  | AAGAGAAGGAGAAGAAGACG |  |
|  |  |  |  |  |  |
| CA_miR5021 | ToLCNDV_RE_RVA | 1644 | -13.4 | AGTGCTGCATTTTTTTCTTC | 7.17E-02 |
|  |  |  |  | \|\|:\| \|:\|\|::\|\|\|\|\| |  |
|  |  |  |  | AAAAGATGAAGAAGGAGAAG |  |
|  |  |  |  |  |  |
| CA_miR5665 | ToLCNDV_RE_RVA | 2222 | -16.6 | GGCCGTCTGCT-GACCACCAC | 2.72E-01 |
|  |  |  |  | \|\| \|\| \|\| \|\|\|\|\|\|\| |  |
|  |  |  |  | AAGGGAGTAGAACAGGTGGTG |  |
|  |  |  |  |  |  |
