# Supplementary figures and images for: Predicting candidate miRNAs for targeting begomovirus to induce sequence-specific gene silencing in chilli plants
Source: Front Plant Sci. 2024 Sep 23;15:1460540. doi: 10.3389/fpls.2024.1460540 (PMC11456425; doi:10.3389/fpls.2024.1460540)

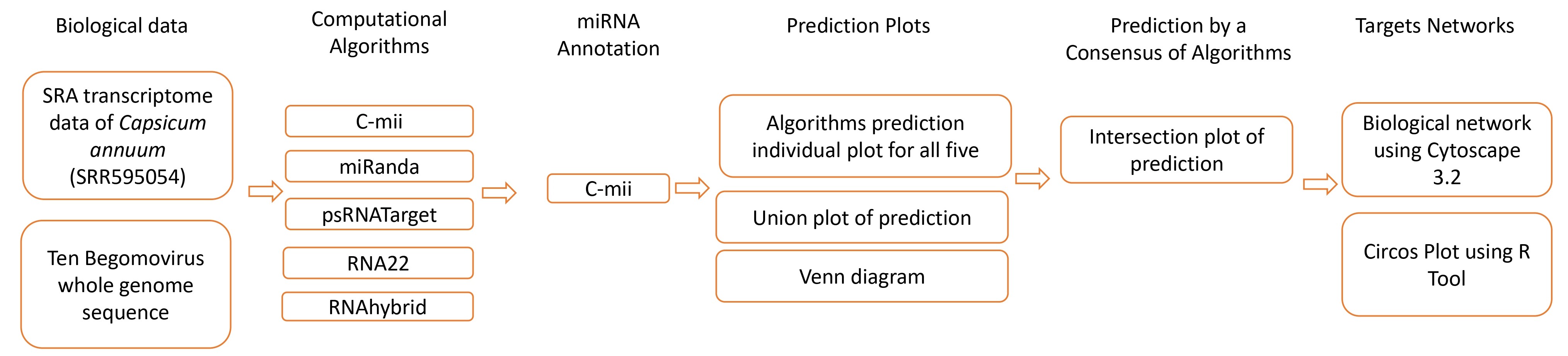

Supplement: Supplementary Figure 1 — A computational methodology for predicting miRNAs offered by hosts using the chilli transcriptome SRA data. Diagram illustrating the many stages of prediction used to examine miRNA targets encoded by chilli found in the genome of begomoviruses. The biological data includes SRA data on chilli, ten begomoviruses. [file Image1.jpeg]

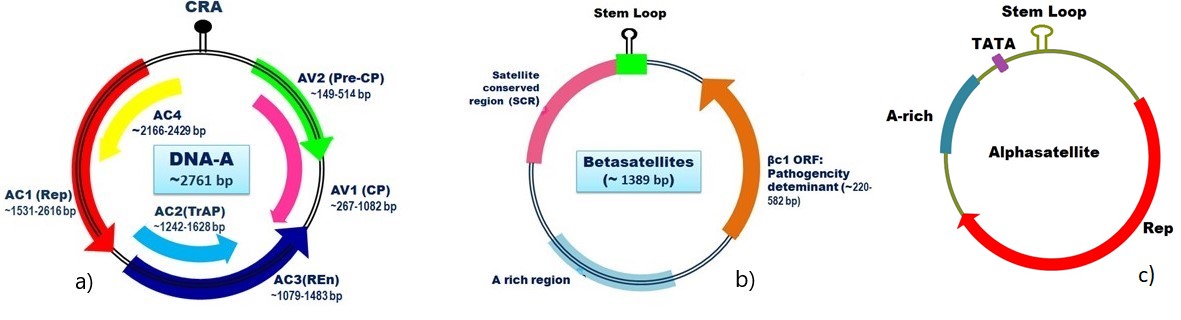

Supplement: Supplementary Figure 2 — Genome organization of begomovirus (A) DNA-A (B) Betasatellite, (C) Alphasatellite [file Image2.jpeg]

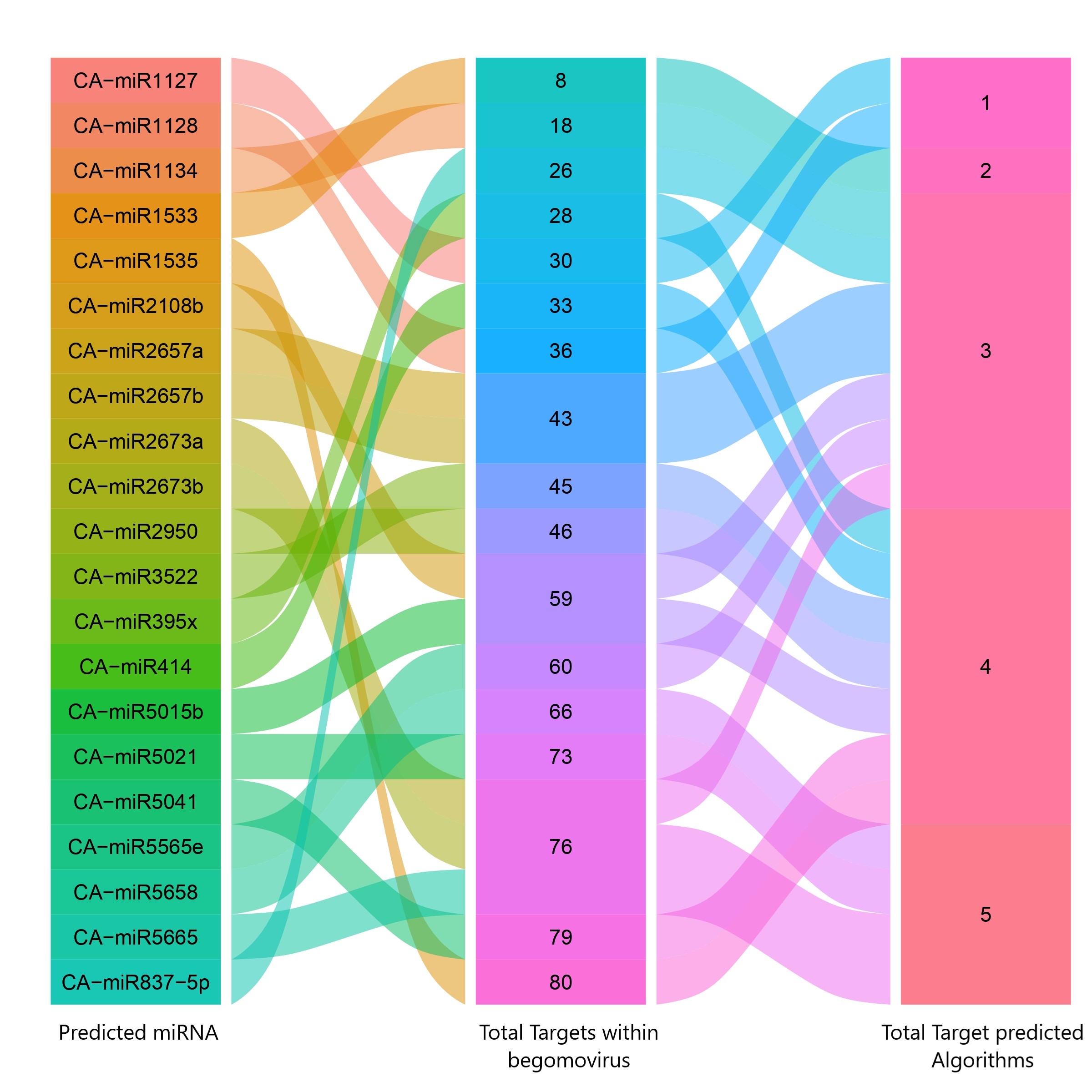

Supplement: Supplementary Figure 3 — Diagrammatic representation of 22 predicted miRNA, its Targets within begomovirus genome and total algorithms validated each predicted miRNA. [file Image3.jpeg]
